# Supplementary material for: Urea use drives niche separation between dominant marine ammonia oxidizing archaea
Source: Nat Commun. 2025 Dec 6;16:10946. doi: 10.1038/s41467-025-67048-1 (PMC12686405; doi:10.1038/s41467-025-67048-1)
Supplement: Supplementary file 1 — Supplementary Information [file 41467_2025_67048_MOESM1_ESM.pdf]

Supplementary Information

Urea use drives niche separation between dominant marine ammonia oxidizing archaea

Authors: Joerdis Stuehrenberg[1‡], Katharina Kitzinger[1,2‡], Jan N. von Arx[1], Jon S. Graf[1], Gaute Lavik[1], Sten Littmann[1], Jana Milucka[1], William D. Orsi[3,4], Sina Schorn[1,†], Daan R. Speth[1,2], Aurèle Vuillemin[3,†], Siqi Wu[1,5], Hannah K. Marchant[1,6,*], Marcel M. M. Kuypers[1]

1 Max Planck Institute for Marine Microbiology, Celsiusstrasse 1, 28359 Bremen, Germany

2 Division of Microbial Ecology, Centre for Microbiology and Environmental Systems Science, University of Vienna, Djerassipl. 1, 1030 Vienna, Austria

3 Department of Earth and Environmental Sciences, Palaeontology & Geobiology, Ludwig-Maximilian-University, Richard-Wagner-Strasse 10, 80333 Munich, Germany

4 GeoBio-Center LMU, Ludwig-Maximilians-Universität München, Munich, Germany

5 State Key Laboratory of Marine Environmental Science, College of Ocean and Earth Sciences, Xiamen University, Xiang'an South Road 4221, 361102 Xiamen, Fujian, China

6 MARUM - Centre for Marine Environmental Sciences University of Bremen, Leobener Strasse 8, 28359 Bremen, Germany

† Current addresses:

Sina Schorn: Department of Marine Sciences, University of Gothenburg, Gothenburg, Sweden;

Aurèle Vuillemin: GFZ Helmholtz Centre for Geosciences Potsdam, Section Geomicrobiology, Telegrafenberg, 14473 Potsdam

‡ Equal contribution

*Corresponding author: hmarchan@mpi-bremen.de

Contents

Supplementary Methods
Supplementary Notes
Supplementary Tables 1, 2, 3
Supplementary References

Supplementary Methods

Database construction for the BLAST Score Ratio approach

The species representatives of the genome taxonomy database (GTDB[1], v207, 65,703 genomes) and the representatives of the operational taxonomic units (OTUs) of the genomic catalog of earth's microbiomes (GEM[2], 45,599 genomes) were dereplicated at 96% average nucleotide identity (ANI) over a minimum of 50% aligned fractions using fastANI[3] (v1.32), for a total of 78,768 dereplicated genomes. Gene calling on this dereplicated set of genomes was done using prodigal[4] (v2.6.3) as implemented in Anvi'o[5] (v7.1). The resulting 200,158,775 predicted amino acid sequences in all genomes were combined and used for marker gene database construction. Custom databases for each of the three genes (*amoA, ureC, dur3*) were compiled by identifying copies of all three genes in all genomes available from the genome taxonomy database (GTDB v207)[1] and the Genomic catalog of Earth's microbiomes (GEM)[2] (Supplementary Data 3). To do so, we used a BLAST score ratio (BSR) approach[6], as previously described[7]. In brief, for each gene a seed dataset or sequence was chosen. For *ureC* and *amoA* the matches retrieved using the online NCBI CDART[8] tool were manually curated and the resulting sets of 7,638 (UreC) and 370 (AmoA) sequences were used as seed datasets, and for Dur3 the sequence of characterized protein from *Ca.* Nitrosopelagicus brevis (PTL88460.1) was used as a seed. Subsequently, the full protein complement of the combined GTDB and GEM genomes (hereafter: "GTDB/GEM proteins") was used to search against the seed using DIAMOND[9] (v2.0.14). The alignment scores of the hits were compared to the theoretical maximum score of a self-hit. The initial custom databases were built under GTDB v202, by using these as seed the databases were updated to GTDB v207 using the procedure described above. Sequence selection is shown in Supplementary Fig. 7a. Scripts for database construction and the three gene databases are available at https://github.com/dspeth/aoa_urea.

The obtained GEM/GTDB genes then served as reference databases for AmoA(and particulate methane monooxygenase subunit A - PmoA, due to the high sequence similarity to AmoA), UreC and Dur3 and consisted of 919, 16,587 and 45 sequences, respectively. The *amoA* gene was present in all marine AOA (class: Nitrososphaeria) reference genomes as a single copy gene; the only AOA genome with two copies of *amoA* is the terrestrial *Nitrosotalea okcheonensis*[10]. Seven reference GEM/GTDB AOA genomes contained two *ureC* copies, but all representatives of the marine *Nitrosopumilus* and '*Ca*. Nitrosopelagicus' genomes harbored

one *ureC* copy. The *dur3* gene was present as a single copy gene in all but one GEM/GTDB AOA genome.

Phylogenetic differentiation of Nitrosopelagicus *WCA and* Nitrosopelagicus *WCB*

AmoA is one of the most frequently amplified genes from environmental samples, including the marine environment[11]. Marine *amoA* sequences are frequently split between two AOA ecotypes, with "Water Column A" (WCA) or "shallow" ecotypes dominating epipelagic waters, and the phylogenetically divergent "Water Column B" or "deep" ecotypes dominating deeper waters e.g.[12-14]. The association of these ecotypes to specific AOA genera is, however, often inconsistent. WCA sequences have mostly been associated with the '*Ca*. Nitrosopelagicus' genus[15], with some studies also including the genus *Nitrosopumilus*, while WCB sequences have not been linked to an AOA genus. Recently, Alves and colleagues[11] unified AOA *amoA* phylogeny. They showed that the majority of marine AOA belong to the clades NP-ε-2 (*Nitrosopelagicus* WCA; cultured representative *Ca.* Nitrosopelagicus brevis) and NP-α-2.2.2.1 (WCB, no cultured representative). The third, less abundant clade NP-ɣ contains *Nitrosopumilus* and *Nitrosarchaeum* and is more prevalent in coastal-estuarine settings[11].

In our analyses, we calculated phylogenetic trees of the retrieved environmental *amoA* sequences, *amoA* reference sequences from Alves et al.[11] and Francis et al.[16], and *amoA* sequences from AOA genomes present in the GTDB[1] and Genomic catalog of Earth's microbiomes (GEM)[2] databases (Supplementary Fig. 10). We observed that *amoA* sequences from GTDB/GEM genomes classified as genus *Nitrosopelagicus* (Supplementary Fig. 9, Supplementary Data 6) clustered with both WCA and WCB reference *amoA* sequences (Supplementary Fig. 10). This indicates that WCA and WCB AOA are indeed both affiliated with the genus *Nitrosopelagicus* (with clear separation in *amoA* sequence similarity and habitat distribution), which we, for the purpose of this manuscript, separated in *Nitrosopelagicus* WCA and WCB (Supplementary Data 6).

We also delineated *Nitrosopelagicus* WCA and *Nitrosopelagicus* WCB on a 16S rRNA gene level, with the aim of designing specific 16S rRNA targeted probes for both groups. We compiled a 16S rRNA gene tree, containing 16S rRNA gene sequences from the GTDB and GEM MAGs, as well as 16S rRNA sequences from SILVA (v138.1, criteria: *Nitrosopumilales*, sequence length >1,399 nucleotides (nt), sequence quality >90, pintail quality >90) (Supplementary Fig. 8). 16S rRNA genes from *Nitrosopelagicus* WCA GTDB/GEM genomes cluster with SILVA 16S rRNA reference sequences defined as "*Nitrosopelagicus*", while 16S

rRNA genes from *Nitrosopelagicus* WCB GTDB/GEM genomes do not have a SILVA genus assignment (Supplementary Fig. 8, Supplementary Data 6). Overall, *Nitrosopelagicus* WCA, WCB and *Nitrosopumilus* form coherent clades across phylogenetic analyses, from *amoA* to 16S rRNA gene to genome trees.

cloneFISH

Since no *Nitrosopelagicus* WCA or WCB cultures were available to optimize formamide concentrations for the newly developed (CARD-)FISH probes, we conducted CloneFISH[17] using plasmid vectors (pet-23a(+)) carrying representative full length 16S rRNA gene sequences for *Nitrosopelagicus* WCA and WCB (16S rRNA gene insert accession number *Nitrosopelagicus* WCA: HQ338108.1; *Nitrosopelagicus* WCB: GCA_013390375; obtained from GenScript, Rijswijk, Netherlands).

The plasmids were transformed into the chemically competent *E. coli* JM109(DE3) by the heat shock method[18]. In brief, 5 ng plasmid were added to a 50 µL *E. coli* aliquot and incubated for 30 minutes on ice. Subsequently, the cells were heat shocked at 42°C for 45 seconds and again put on ice for 2 minutes. Then, cells were gently mixed with 800 µL RT-SOC-Medium[19] and incubated at 37°C for 1h while gently shaking. Cells were spread onto LB plates containing 100 µg mL^{-1} ampicillin (Sigma-Aldrich, Taufkirchen, Germany)[19] and incubated overnight at 37°C. Then, clones were picked and transferred to fresh LB-ampicillin plates and incubated at 37°C for about 8h.

A standard PCR was performed to check for the presence of the desired plasmid in the *E. coli* cells. Single colonies were picked and directly transferred into the PCR master mix (20 µL), which contained 2 µL bovine serum albumin (BSA, 3 mg), 2 µL dNTPs (10 mM), 1.6 µL Taq buffer (10x), 50 µM each T7 forward and reverse primer, 0.2 U µL^{-1} Taq polymerase and 13 µL PCR water. The PCR was done with an initial denaturation at 95°C for 10 min, and 35 cycles of 60 sec 95°C denaturation, annealing for 30 sec at 55°C and elongation for 90 sec at 72°C. The PCR products were evaluated by agarose gel electrophoresis (1.2% LE agarose gel in 1x TAE and 7 µL ml^{-1} SYBR safe) and were Sanger-sequenced using BigDye dye terminator kit. In brief, PCR products were desalted and purified with Sephadex G-50 + HPLC-water by centrifuging the sample for 5 min at 910 rpm. Subsequently, 4 µL master mix (0.8 µL Big Dye, 1.2 µL 2.5 x reaction buffer, 5 pmol L^{-1} T7_forward primer) were added to 1 µL of desalted PCR product. Annealing temperature was 55°C with 99 cycles. Then, the sequencing reaction was purified again using Sephadex G-50 + HPLC-water and sequenced using the

BigDye Terminator v.3.1 sequencing kit (Thermo Fisher Scientific). The obtained sequences were compared against the vector sequences using blastn[20] and the presence of the probe binding site (with zero mismatches) was confirmed.

Clone-FISH was performed as previously described[17]. All liquid LB medium contained 100 µg mL^{-1} ampicillin as selective pressure. In brief, 5 mL LB medium were inoculated at 37°C overnight with a single picked clone containing the plasmid. Overnight cultures were diluted 1:20 with LB medium and incubated at 37°C to an OD_{600} of 0.3 to 0.4. To induce transcription of the cloned 16S rRNA gene, Isopropyl-β-D-thiogalactopyranoside (IPTG, 1mM final concentration) was added and cells were incubated for 1 hour at 37°C, then, chloramphenicol (170 µg mL^{-1} final concentration) was added for maximizing RNA content of the cell, and cells were incubated for 4 h at 37°C. Finally, cells were fixed in 1% paraformaldehyde (PFA), washed and stored in PBS:ethanol (2:3) at -20°C[21].

Formamide Concentration Series

To determine optimal hybridization conditions, a formamide concentration series was carried out for each newly designed probe and clone[22]. DOPE-FISH was performed on the E. coli clones transcribing *Nitrosopelagicus* WCA and WCB 16S rRNA genes as described previously[23]. Fixed cells were washed twice in ddH_2O by centrifugation and resuspension. Then, 5 µL of 1:100 cell dilution was dried on microscope slides and dehydrated with an ethanol series (50% EtOH, 80% EtOH and 96% EtOH, 3 min each). After air drying, 10 µL of hybridization buffer containing 1 µL of probe and competitors (final probe concentrations of 0.5 pmol $µL^{-1}$ each) was applied and mixed on the microscope slides. The slide was incubated at 46°C in a 50 mL centrifuge tube that contained a tissue soaked in hybridization buffer. Each slide was incubated for 2 to 3 h. Subsequently, slides were transferred to pre-warmed (48°C) washing buffer corresponding to the respective formamide concentration for 10 min. Lastly, the slides were dipped into ice-cold ddH_2O and dried with compressed air. Each slide was incubated and washed for the exact same amount of time.

The formamide concentration series for the *Nitrosopumilus* specific probe Npum_229 was performed on a fixed (3% PFA for 1h at room temperature, stored in 1:1 EtOH:PBS) culture of *Nitrosopumilus adriaticus* NF5[24] with a CARD-FISH protocol, since attempts to generate a formamide concentration series with the DOPE-FISH probes failed, due to low signal intensity. 5 µL of the cell suspension was dried on microscope slides and then dehydrated with an

ethanol series (50% EtOH, 80% EtOH and 96% EtOH, 3 min each). Subsequently, the CARD-FISH protocol described in the main manuscript methods section was applied.

For epifluorescence microscopy, cells were embedded in a CitiFlour Vectashield (4:1) mixture. Pictures were acquired with an epifluorescence microscope (Axioplan 2, Zeiss) and processed using Zeiss ZEN blue (v.3.2). The exposure time was determined at 10% or 15% formamide concentration and kept constant for all formamide concentrations of the respective probe. Because of high signal intensity, the exposure time for the probe Npum_229 was set at 30%. For each formamide concentration, five pictures were taken at randomly selected fields of view and analyzed in daime (v.2.2.3)[25]. The automatic segmentation according to edge detection was used for object identification. The optimal formamide concentrations for each newly designed probe can be found in Supplementary Table 2 and Supplementary Fig. 6.

Phylogenetic analyses of key functional genes

Assembled *amoA* and *ureC* sequences from the Gulf of Mexico have been previously published[26].

The Angola Gyre metagenomes were individually assembled using SPAdes v.3.13.0 (-k 21,33,55,77)[27]. For the Black Sea, *amoA* and *ureC* genes were obtained by extracting all metagenomic and metatranscriptomic AOA reads identified through the BSR approach and assembling those into contigs using IDBA (v.1.1.3, IDBA-UD)[28]. Open reading frames (ORF) in both assemblies (IDBA-UD, SPAdes) were predicted using Prodigal v.2.6.3[4]. For the Angola Gyre assembly, we used hmmsearch[29] to identify *amoA* and *ureC* genes (Pfam hmm model PF12942.9 for AmoA, --cut_ga; TIGRFAM hmm model TIGR01792.1, --cut_nc for UreC).

For all environments (Gulf of Mexico, Black Sea and Angola Gyre), we retained *amoA* sequences >340 bps and UreC sequences >380 aa and clustered them at 95% identity[30] (Supplementary Data 6).

Phylogenetic analyses of metagenome assembled genomes

Metagenome assembled genomes (MAGs) from the Gulf of Mexico have been previously published[26]. For the Angola Gyre metagenomes, no MAGs could be binned, likely due to the shorter than expected length of the metagenomic reads (average length 123 bps, Supplementary Data 3). For the Black Sea, trimmed metagenomic reads were assembled using MEGAHIT (v.1.2.9)[31]. The quality of the assembly was assessed by mapping the reads

onto the assembly with CoverM (v.0.6.1)[32]. The read recruitment was between 71 to 81% in all the samples (see Supplementary Table 8). Three samples (5158_A, 5158_D, 5158_H; Supplementary Data 3) were processed individually in SqueezeMeta (v.1.5.1) providing the MEGAHIT assembly (minimum contig length = 1,000)[33].

For differential coverage binning of the Black Sea datasets, all the reads were mapped onto the individual assemblies of the three samples that were also chosen for the SqueezeMeta (v1.5.1) pipeline with CoverM (v0.6.1)[32,33]. The coverages of all contigs were used to create differential coverage plots, where the genes of interest (*amoA* & *ureC*) were highlighted. This was done using the gene prediction generated by Prodigal during the SqueezeMeta pipeline[4,33]. To pre-select subsets for refining the metagenome assembled genomes (MAGs), broad contig clusters (containing a maximum of 10,000 to 20,000 contigs) surrounding the genes of interest (i.e. contigs with similar differential abundance patterns across samples) were selected. These subsets were analyzed in a workflow using anvi'o (v.7.1), which mapped the reads onto the contigs[34]. Ultimately, MAGs were refined and selected interactively in anvi'o (v.7.1) from a dendrogram with hierarchical clustering according to differential coverage and sequence composition[34]. MAGs assigned to be *Nitrosopumilus sp.* had high completeness (90 to 94%) and low redundancy (5 to 8 %) already at this step of the analysis. However, MAGs assigned to be *Ca.* Nitrosopelagicus sp. had a lower completeness (65 to 80%) and higher contamination (8 to 15%). To improve the quality of the MAGs, the initial selection of contigs was conservative with the lowest contamination possible. Then the reads were iteratively (20 times) elongated by mapping them onto the contigs in the bin with a minimum read identity of 98% and a minimum of aligned bases per read of 80%. More specifically, this was done by aligning the reads, belonging to the contigs within the incomplete MAGs, using minimap2 (v.2.24-r1122)[35,36]. Subsequently, the alignments were sorted using samtools (v.1.16.1) and filtered using CoverM (v.0.6.1)[32,37]. The mapping reads were extracted using seqkit (v.2.3.0) and separately assembled using SPAdes (v3.15.3)[38,39]. This assembly was then used in the next iteration. The assembly from each iteration was checked for completeness and contamination using CheckM (v.1.2.2) to choose the highest quality assembly[40]. Lastly, from this assembly, the MAG was defined using the interactive interface in anvi'o (v.7.1)[34]. This ultimately led to six highly complete and low contaminated MAGs from the Black Sea, three belonging to the genus *Nitrosopumilus* and three belonging to the genus *Ca.* Nitrosopelagicus (Supplementary Table 8, Supplementary Fig. 9).

Key Equations

Breakdown of ^{15}N-urea to ^{15}N-ammonium was obtained by measuring combined ammonium and nitrite ($^{15}N_{ammonium+nitrite}$) by hypobromite conversion[41] and by measuring nitrite ($^{15}N_{nitrite}$) by acetic azide conversion[42] and ultimately subtracting the measured concentrations.

$$^{15}N_{ammonium} = {}^{15}N_{ammonium+nitrite} - {}^{15}N_{nitrite} \quad (1)$$

Detection limits for bulk oxidation rates (calculated per treatment and cruise) were calculated using the average measured standard error of the slope of rates in the range from 0 to 2 times the minimum significant measured rate ($sd_{minrates}$), multiplied by the t value for p = 0.05 ($t_{0.05}$)[43,44].

$$LOD_{rates} = sd_{minrates} \times t_{0.05} \quad (2)$$

RPKM values for each sequence in the AOA *amoA*, *ureC* and *dur3* database in our metagenome and metatranscriptome were calculated:

$$RPKM = numReads \div (geneLength \times 10^{-2} \times totalNumReads \times 10^{-6}) \quad (3)$$

where numReads is the number of reads mapping to the respective reference sequence in one sample, geneLength is the average gene length in nucleotides of the respective gene (651, 1716 and 1980 nt for AOA *amoA*, *ureC* and *dur3*, respectively), and totalNumReads is the total number of reads per sample.

The limit of detection (LOD_{cell}) for the $^{15}N/(^{14}N + ^{15}N)$ enrichment was calculated for each nanoSIMS filter:

$$LOD_{cell} = mean_{background} + 3 \times sd_{background} \quad (4)$$

where $mean_{background}$ is the averaged $^{15}N/(^{14}N + ^{15}N)$ enrichment of the filter and $sd_{background}$ is the corresponding standard deviation.

The cellular ^{15}N-atom% excess was calculated

$$^{15}\text{N-atom\%excess}_{\text{cell}} = (\text{enrichment}_{15\text{N}/(15\text{N} + 14\text{N})} - 0.0037) \times 100 \quad (5)$$

as in ref. 45, where $\text{enrichment}_{15\text{N}/(15\text{N} + 14\text{N})}$ refers to the measured single cell enrichment.

Growth rates for significantly enriched single cells were calculated

$$\text{Growthrate}[\text{d}^{-1}] = \log_2[^{15}\text{N-atom\%excess}_{\text{inc}} \div (^{15}\text{N-atom\%excess}_{\text{inc}} - {}^{15}\text{N-atom\%excess}_{\text{cell}})] \times 1 \div \text{time} \quad (6)$$

as in ref. 46, where ^{15}N-atom%excess$_{\text{inc}}$ and ^{15}N-atom%excess$_{\text{cell}}$ refers to the incubation substrate atom% excess (calculated analogously to the single cell atom% excess values), and the single cell atom% excess, respectively.

Supplementary Notes

Supplementary Note 1

Between the three sampled environments, some details differed in the conducted incubation experiments to quantify ammonia and urea oxidation rates, as described in the methods section of the main text. However, given that urea use has previously been reported to increase towards oligotrophic regions using several different experimental setups, and conducted by different authors (e.g. refs.[13,47]), we are confident that minor differences in protocols did not impact the outcome of our experiments, or our conclusions.
^{15}N-tracer amendments were adjusted to reflect the overall productivity of the respective environment. Although all of the measured rates are potential rates due to the addition of ^{15}N-tracers, the short length of the incubations (< 24h) and the linearity of the rates from the beginning of the incubation on, without lag phase, indicate that the ammonia oxidizers were actively using ammonia and urea as energy and N-sources *in situ*.
Within each cruise, only treatments were compared (i.e. ammonia and urea oxidation) that received the same amendments in regard to oxygen and DIC. Oxygen availability plays a key role for AOA activity (as it is required for activation of ammonia into hydroxylamine and as terminal electron acceptor), and oxygen affinities of AOA are exceptionally high (nM range[48]). Across our experiments, oxygen was adjusted to reflect *in situ* conditions, however, we anticipate that even Black Sea samples from depths without detectable oxygen contained some residual oxygen as even careful handling introduces oxygen contamination[49]. The linearity of our rates, also in low oxygen samples, indicates that oxygen was present at higher concentrations than required for AOA activity.

Supplementary Note 2

AOA may utilize urea as an energy and N-source both directly (via AOA-encoded ureases), or indirectly via cross-feeding on ammonia released by other urease-encoding community members expressing urease. Differentiation of these two scenarios in ^{15}N-tracer experiments can be achieved by conducting two parallel experiments, one with only ^{15}N-urea, and one with ^{15}N-urea, and additionally a large unlabeled ammonium pool (ammonium pool incubations)[26].

In the ^{15}N-urea only incubations, breakdown and release of ^{15}N-ammonium from ^{15}N-urea by other microorganisms would progressively increase the amount of ^{15}N in the extracellular ammonium pool, which would lead to exponential production of ^{15}N-nitrite from ammonia oxidation over time. In ammonium pool experiments, both ^{15}N-ammonium release from ^{15}N-

urea into the surrounding seawater can be quantified, and due to the strong dilution in the unlabeled ammonium pool, any released ^{15}N-ammonium is less likely to be further oxidized. Thus, the rate of ^{15}N-nitrite production would be strongly decreased and any observed ^{15}N-nitrite in these experiments would likely stem from direct urea utilization by AOA.

In the Gulf of Mexico, where most detected and transcribed ureases were affiliated with Proteobacteria (Supplementary Fig. 5), there was evidence of indirect utilization - i.e. formation of ^{15}N-ammonium which is subsequently taken up and oxidized by the AOA[26]. However, we can largely rule out that indirect urea-oxidation was significant in the Angola Gyre and Black Sea. We only measured minute production of ^{15}N-ammonium in the pool incubations, and ^{15}N-nitrite production rates were highly similar between incubations without and with added ammonium pool. Analogously, if indirect utilization played a major role in N-assimilation, we would expect to see a strong decrease in ^{15}N-assimilation in the urea incubations with $^{14}NH_4^+$ compared to those without. However, *Nitrosopelagicus* did not show decreased urea assimilation rates in presence of ammonium.

Supplementary Note 3

For Angola Gyre samples, we could unfortunately not obtain sufficiently strong CARD-FISH signals to visualize AOA cells in our PFA fixed samples (fixation and filtration as described in the main text for the Black Sea samples), despite trying to optimize the CARD-FISH protocol. We hypothesize that this is likely due to a combination of the small cell size of open ocean AOAs and a potentially low cellular ribosome content (and thus signal intensity) stemming from the much lower per cell ammonia and urea oxidation activity compared to AOA in the Black Sea and the Gulf of Mexico. This precluded both direct cell counts and NanoSIMS analyses for Angola Gyre AOA.

Therefore, to estimate AOA abundance in the Angola Gyre and compare it to the other sampled environments, we performed total microbial cell counts after DAPI staining of PFA fixed, filtered samples, on an epifluorescence microscope.

We then combined these total cell counts per milliliter with the relative abundance information from 16S rRNA gene analyses based on metagenome reads[50] (Supplementary Data 1), assuming one rRNA gene copy per cell, to estimate total AOA abundance in the Angola Gyre samples. We expect this approach to be conservative, as many microbes may possess more than one rRNA gene copy per genome.

Supplementary Figures

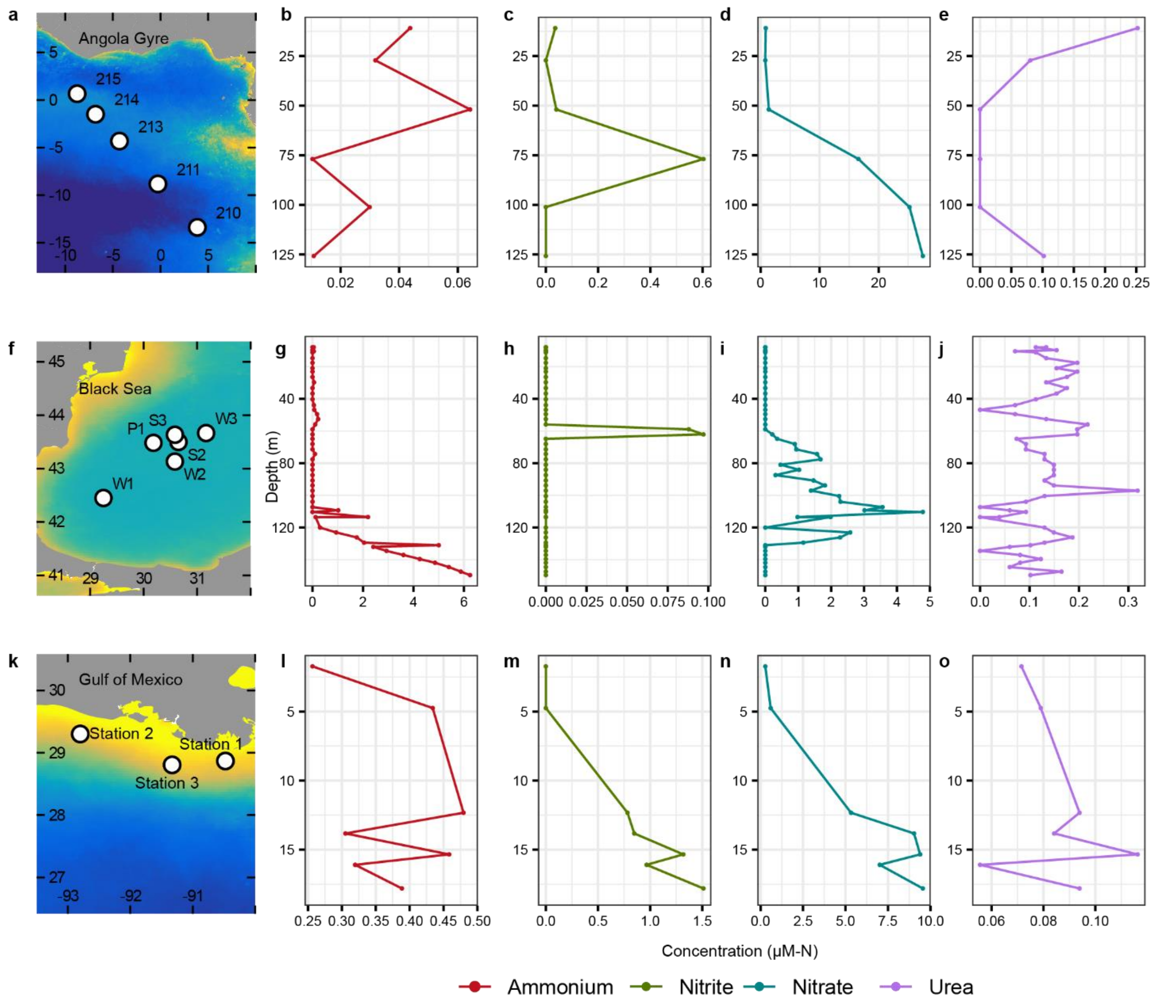

Supplementary Fig. 1: Depth distribution of nutrients in the Angola Gyre, the Black Sea and the Gulf of Mexico. Station maps of EreBUS cruise M148/2, which crossed through the Angola Gyre (a), Poseidon cruise POS539 in the Black Sea (f), Pelikan cruise PE17-02 in the Gulf of Mexico (k), surface chlorophyll *a* values (averaged over the time period between January 1st, 2016 and December 31st, 2019[75], (doi: 10.5067/AQUA/MODIS/L3M/CHL/2022). Representative depth profiles of (b, g, l) ammonium-N, (c, h, m) nitrite-N, (d, i, n) nitrate-N, (e, j, o) urea-N from Gulf of Mexico station 1, Black Sea station S3 and Angola Gyre station 210. Note the different x- and y-axis scales. Experimental stations are indicated by station number. Panel a, f, k were generated using QGIS v.3.32.2, panels b-e, g-j and l-o using RStudio (v.2023.12.1) including ggplot2 (v.3.5.1). Source data are provided as a Source Data file.

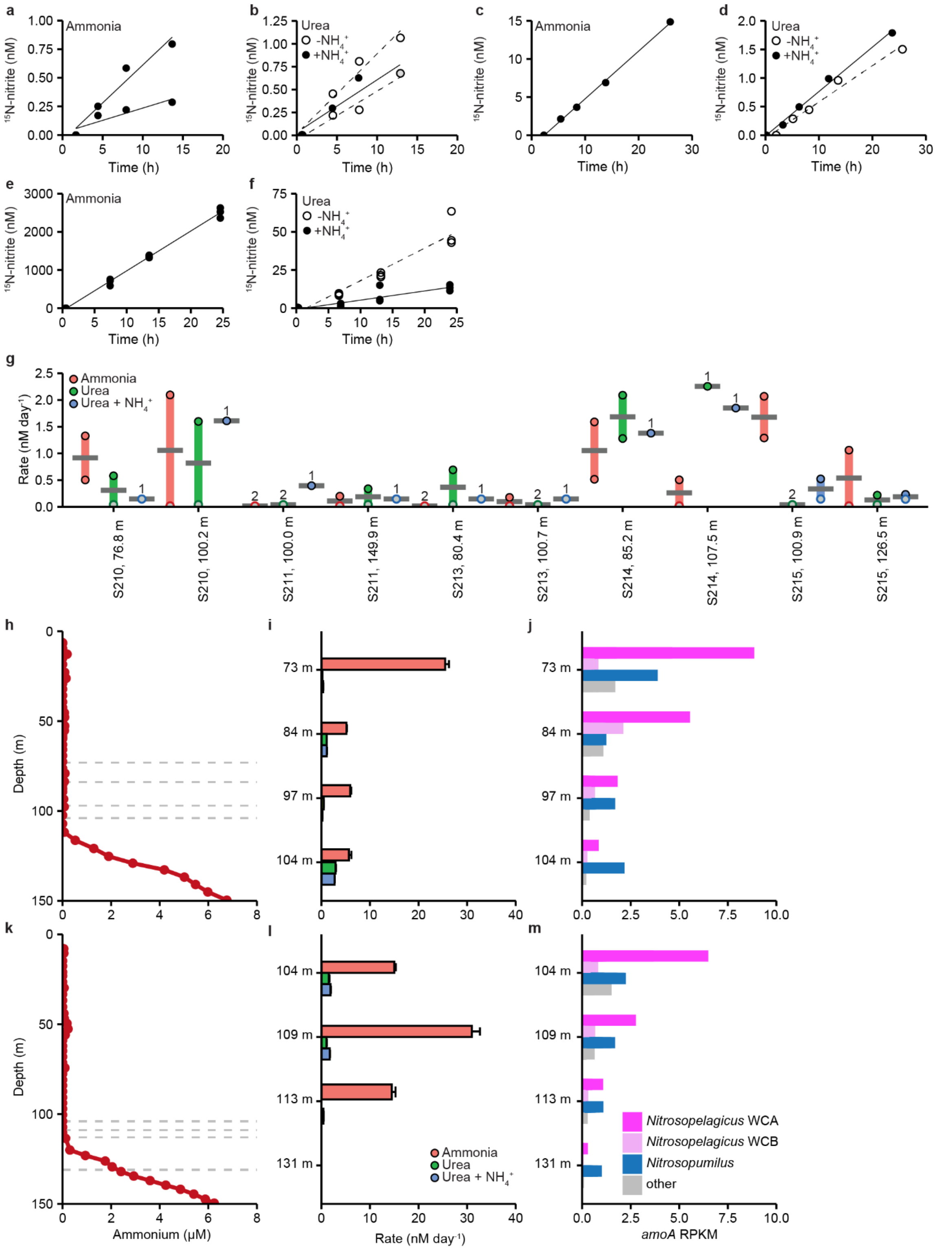

Supplementary Fig. 2: Linearity of oxidation rates (a-f) and individual replicate oxidation rates for the Angola Gyre (g) as well as rates and AOA community composition over

depth in the Black Sea (h-m). ^{15}N-nitrite concentration over time after addition of ^{15}N-ammonium and ^{15}N-urea without (open circles) and with (filled circles) added ^{14}N-ammonium in the Angola Gyre at Station 214, 85 m (a, b, respectively), in the Black Sea at station S3, 105 m (c, d, respectively) and in the Gulf of Mexico at station 2, 14 m (e, f, respectively). Data points in the Angola Gyre are biological duplicates, in the Black Sea individual biological replicates that were sacrificed per time point and in the Gulf of Mexico biological triplicates. Lines are linear regressions across all time points. (g) Ammonia and urea (+ ^{14}N-ammonium) oxidation rates for depths below 80 m in the Angola Gyre shown as ranges (colored stripe) with the average over replicates (gray crossbar) and the replicates as circles. Significant rates above the limit of detection are represented by color-filled circles with a black outline, non-significant rates are filled gray with a colored outline and set to the limit of detection. Numbers above single points indicate the amount of measurements. Depth distribution of ammonium in the Black Sea at station W3 (h) and S3 (k). Grey dashed lines indicate incubation depths. Ammonia and urea (+ 14N-ammonium) oxidation rates in the Black Sea from station W3 (i) and S3 (l). RPKM values of *amoA* of the different AOA groups (*Nitrosopelagicus* WCA - magenta, *Nitrosopelagicus* WCB - rose, *Nitrosopumilus* - blue, other AOA - gray) across metagenome samples from incubation depths in the Black Sea station W3 (j) and S3 (m). Source data are provided as a Source Data file.

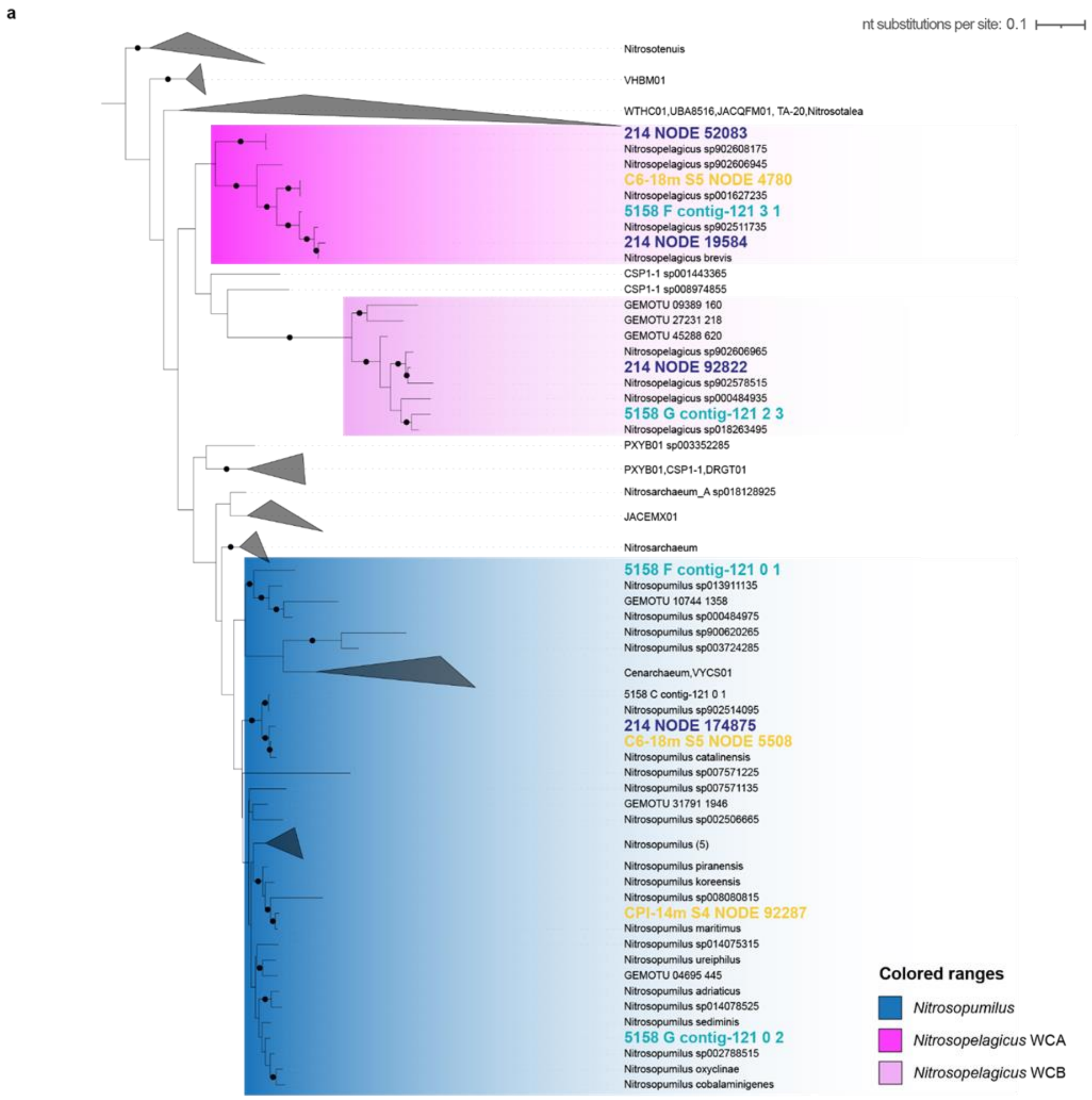

Supplementary Fig. 3: Phylogenetic placement and abundance of obtained AOA *amoA* sequences. *amoA* phylogenetic tree including assembled sequences from all three environments (minimum length 430 bp) and GEM/GTDB reference sequences. All sequences (reference and assembled) were clustered at 95% identity using UCLUST[30]. Phylogenetic tree was calculated using IQ-TREE2[51], model GTR+F+R6, based on a MAFFT (v.7.487)[52] alignment, trimmed using trimal (v1.4.rev15)[53] and re-rooted at *Nitrosotenuis*. Scale bar represents nucleotide substitutions per site. Assembled sequences are colored according to environment (Gulf of Mexico - yellow, Black Sea - turquoise, Angola Gyre - dark blue).

a

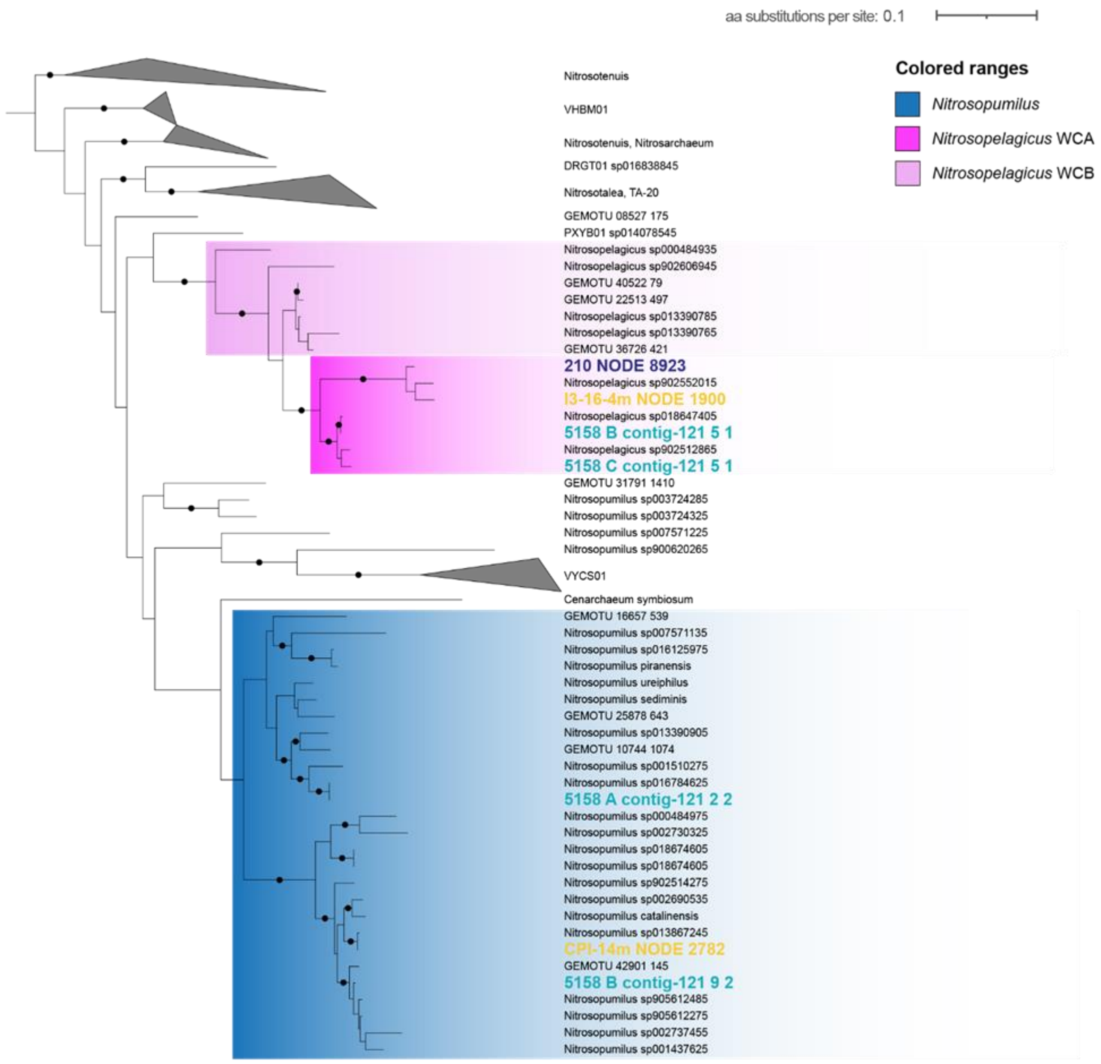

Supplementary Fig. 4: Phylogenetic placement and abundance of AOA UreC sequences in the three different environments. UreC phylogenetic tree including assembled sequences from all three environments (minimum length 380 amino acids, clustered at 95% identity using UCLUST[30]) and GEM/GTDB reference sequences. Phylogenetic tree was calculated using IQ-TREE2[51], model Q.plant+R4, based on a MAFFT (v.7.487)[52] alignment, trimmed using trimal (v1.4.rev15)[53] and re-rooted at *Nitrosotenuis*. Scale bar represents amino acid substitutions per site. Assembled sequences are colored according to environment (Gulf of Mexico - yellow, Black Sea - turquoise, Angola Gyre - dark blue).

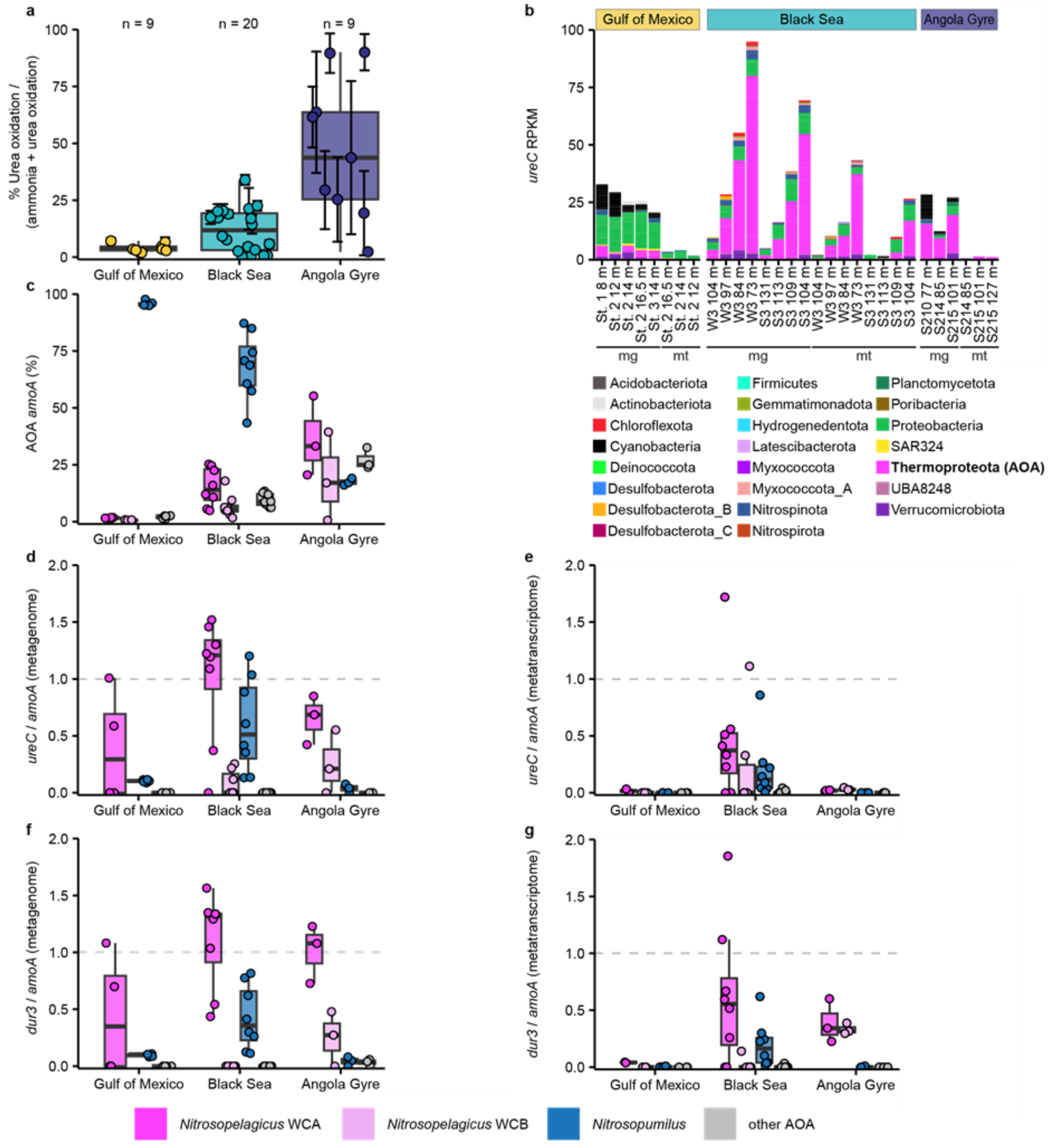

Supplementary Fig. 5: Comparison between urea oxidation rates and key AOA ammonia and urea utilization genes in different environments. (a) Urea oxidation rates as a percent of combined ammonia and urea oxidation rates in the Gulf of Mexico (yellow), the Black Sea (turquoise) and the Angola Gyre (dark blue). Percentages (one value per depth) are calculated for all depths from the Gulf of Mexico and the Black Sea and for depths below 80 m in the Angola Gyre. Non-significant rates were set to the limit of detection (6.3 nM day^{-1} in the Gulf of Mexico, 1 depth below LOD; 0.08 nM day^{-1} in the Black Sea, 3 depths below LOD; 0.04 nM day^{-1} in the Angola Gyre, 2 depths below LOD). Boxplots depict the 25–75% quantile range, with the center line depicting the median (50% quantile); whiskers encompass data points within 1.5× the interquartile range. n = number of depths analyzed per region. (b) RPKM values of *ureC* (phylum level, RPKM > 0.5) in metagenomic and metatranscriptomic samples from

the three environments. (c) Relative abundance (%) of AOA *amoA* assigned to *Nitrosopelagicus* (WCA magenta, WCB rose), *Nitrosopumilus* (blue) and other AOA (gray) across all metagenomic samples in the Gulf of Mexico, the Black Sea and the Angola Gyre. Ratios of *ureC* to *amoA* and *dur3* to *amoA* of the different AOA groups (*Nitrosopelagicus* WCA - magenta, *Nitrosopelagicus* WCB - rose, *Nitrosopumilus* - blue, other AOA - gray) across metagenome (d, f, respectively) and metatranscriptome (e, g, respectively) samples from the Gulf of Mexico, the Black Sea and the Angola Gyre. Gray dashed lines (d, e, f, g) show the 1:1 ratio, indicating equal copy numbers of *amoA*, and *ureC* or *dur3* (i.e. in the Black Sea almost every *Nitrosopelagicus* WCA encoded at least one *ureC* and one *dur3*). Ratios where AOA *amoA* RPKM of one group was below 0.4 for metagenomes, and 0.1 for metatranscriptomes, respectively, are not depicted. Note that Proteobacteria (all four environments) and Cyanobacteria (Gulf of Mexico and Angola Gyre) are additional abundant groups encoding and transcribing *ureC* (b). Source data are provided as a Source Data file.

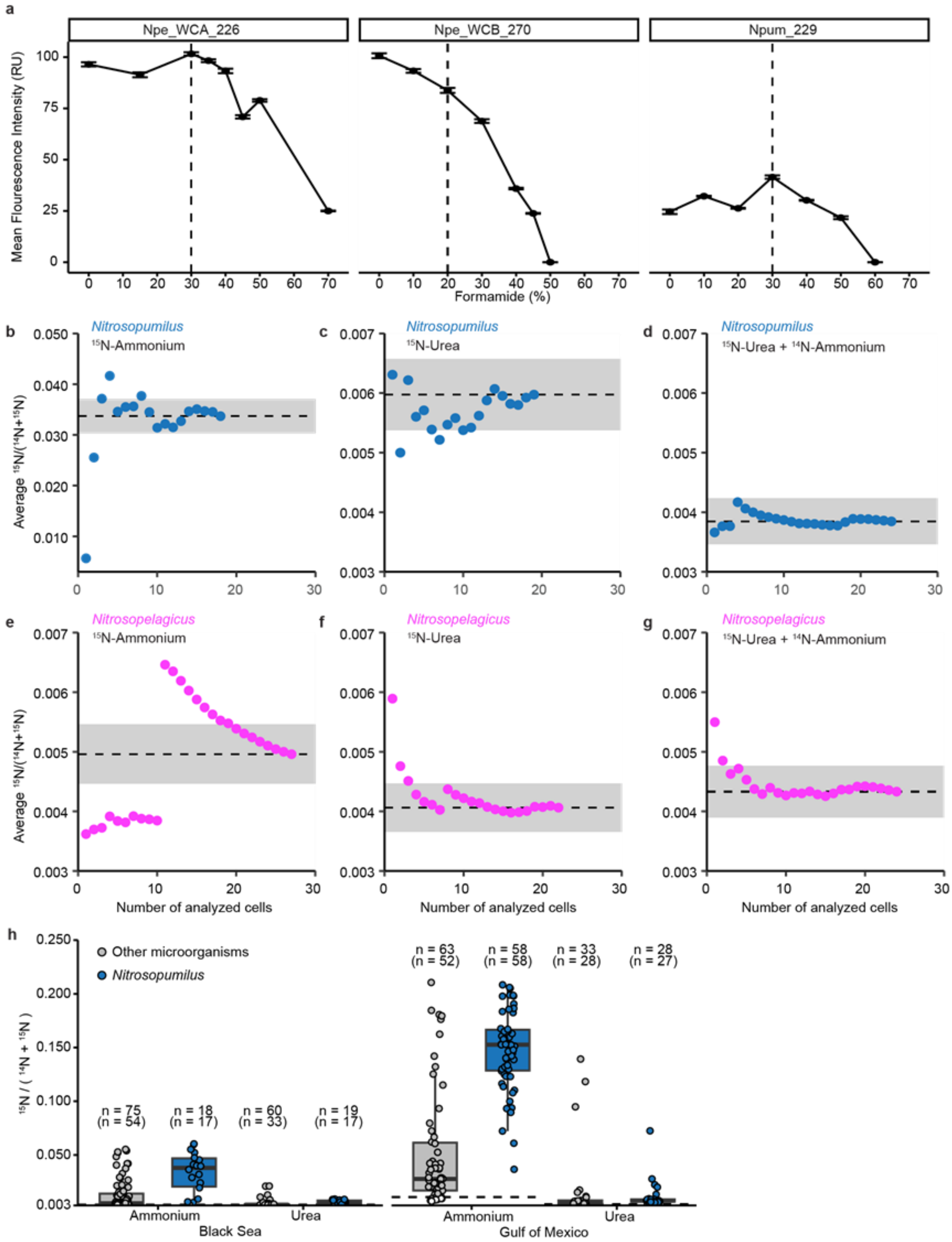

Supplementary Fig. 6: (a) Formamide concentration series for newly designed probes Npe_WCA_226, Npe_WCB_270 and Npum_229. Dashed vertical lines indicate the formamide concentration used for all CARD-FISH experiments. (b-g) Enrichment statistics of *Nitrosopumilus* cells (blue) and *Nitrosopelagicus* cells (magenta) analyzed by NanoSIMS. Dots represent the means of $^{15}N/(^{14}N+^{15}N)$ ratios calculated across randomly subsampled cells. The black dashed line represents the mean across all cells, the light gray area ± 10% of

the mean. Note that the scale for ^{15}N-ammonium in *Nitrosopumilus* is different. The total number of analyzed cells was b) n = 18, c) n = 19, d) n = 24, e) n = 27, f) n = 22, g) n = 24. (h) $^{15}N/(^{14}N + ^{15}N)$ enrichment of *Nitrosopumilus* (blue) and other microorganisms (gray) after incubation with ^{15}N-ammonium and urea in the Black Sea and the Gulf of Mexico. Boxplots depict the 25–75% quantile range, with the center line depicting the median (50% quantile); whiskers encompass data points within 1.5× the interquartile range. Dashed lines represent the limit of detection (10.75×10^{-3}, 4.43×10^{-3}, 4.06×10^{-3}, 3.72×10^{-3} for ammonium and urea in the Gulf of Mexico and the Black Sea, respectively. Cells that were below the limit of detection are filled white. Natural abundance of $^{15}N/(^{14}N+^{15}N)$ is 3.7×10^{-3}. ^{15}N at% enrichment of the substrate pools (ammonium or urea, respectively) was >97%. Three values from the Gulf of Mexico are not depicted (402×10^{-3}, 320×10^{-3} for *Nitrosopumilus* and other microorganisms on ammonium and 326×10^{-3} other microorganism on urea), but were included in all calculations. n = number of measurements per group and treatment with number of cells enriched significantly above the detection limit in brackets. Source data are provided as a Source Data file.

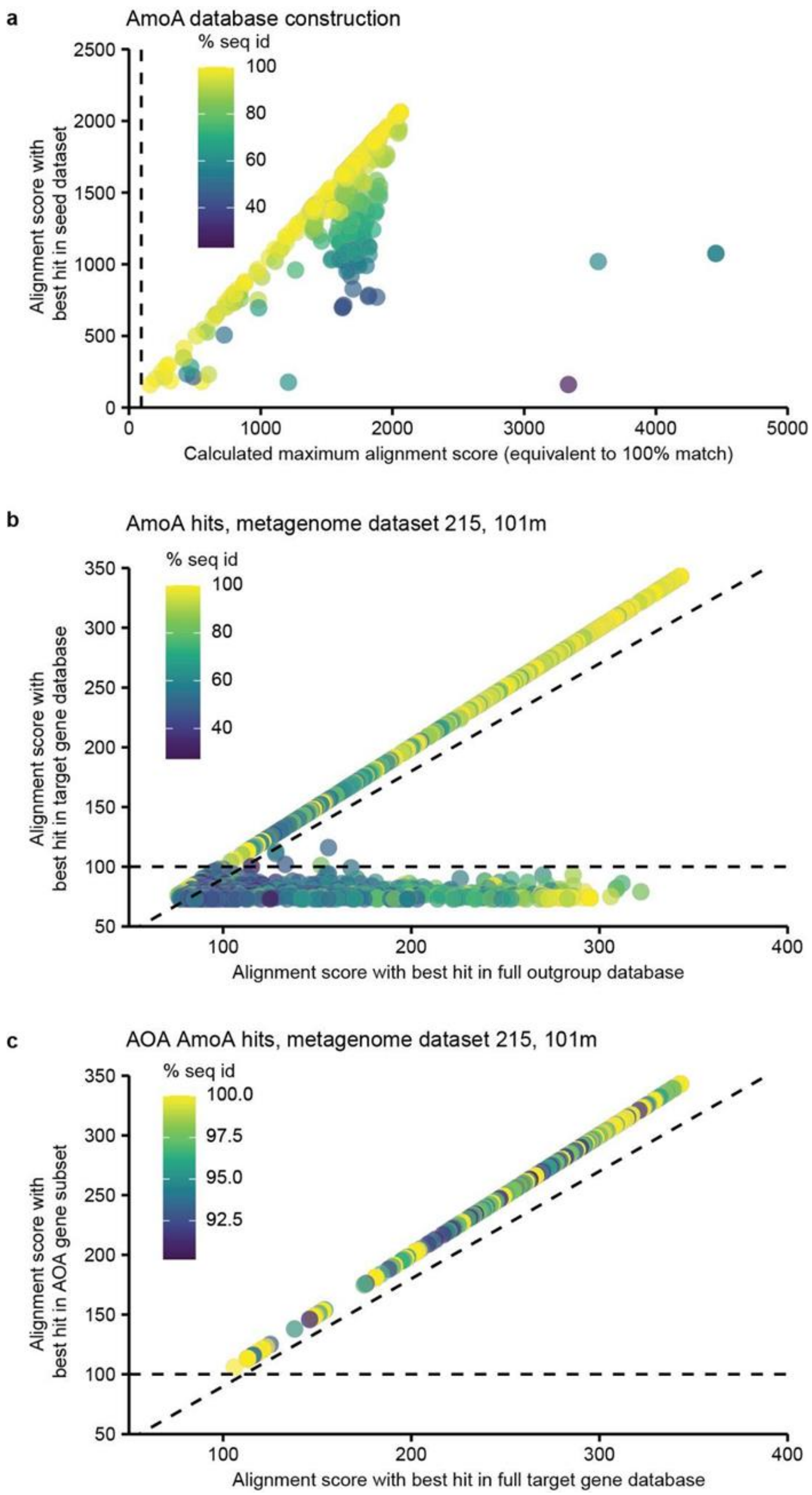

Supplementary Fig. 7: Examples for read trawling approach. (a) AmoA reference database construction. The total protein complement of genomes in the GTDB (v207) species representatives set and the GEM genomes set was dereplicated and used for a DIAMOND blastp search against the previous AmoA reference dataset (v202). For each sequence with a hit against the previous dataset the maximum possible score was calculated. Plot shows the

maximum possible score, which is proportional to sequence length, on the X-axis and the alignment score of the best hit against the seed dataset on the Y-axis. The dashed lines indicate cutoff values chosen in sequence selection. The sequences that do not fall on the 1:1 line are sequences that were added to the reference data between v202 and v207. (b) Station 215, 101 m depth metagenome read trawling against the entire AmoA/PmoA reference database using DIAMOND blastx[9]. To remove false positive hits, while keeping reads resulting from divergent sequences not present in the reference dataset, the score of a hit against the reference dataset was compared to the score of a hit against the GTDB/GEM genomes, using a blast score ratio. Reads with a score of 100 or better against the reference dataset, a blast score ratio of over 0.9 and a sequence identity over 90% were kept as true positive hits. Each dot represents a read, the X-axis represents the score against the full GTDB/GEM protein complement and the Y-axis represents the score against a reference database. The cutoff values are indicated on the plots with dashed lines. (c) Separation of AmoA reads into AOA and non-AOA.The blast score ratio procedure was repeated with only the true-positive reads from the read trawling. A hit against the AOA GTDB/GEM subset was compared with a hit against the complete dataset using blast score ratio. Each dot represents a read, the X-axis represents the score against the full reference dataset (including AOA sequences) and the Y-axis represents the score against the AOA subset database. The cutoff values are indicated on the plots with dashed lines. Source data are provided as a Source Data file.

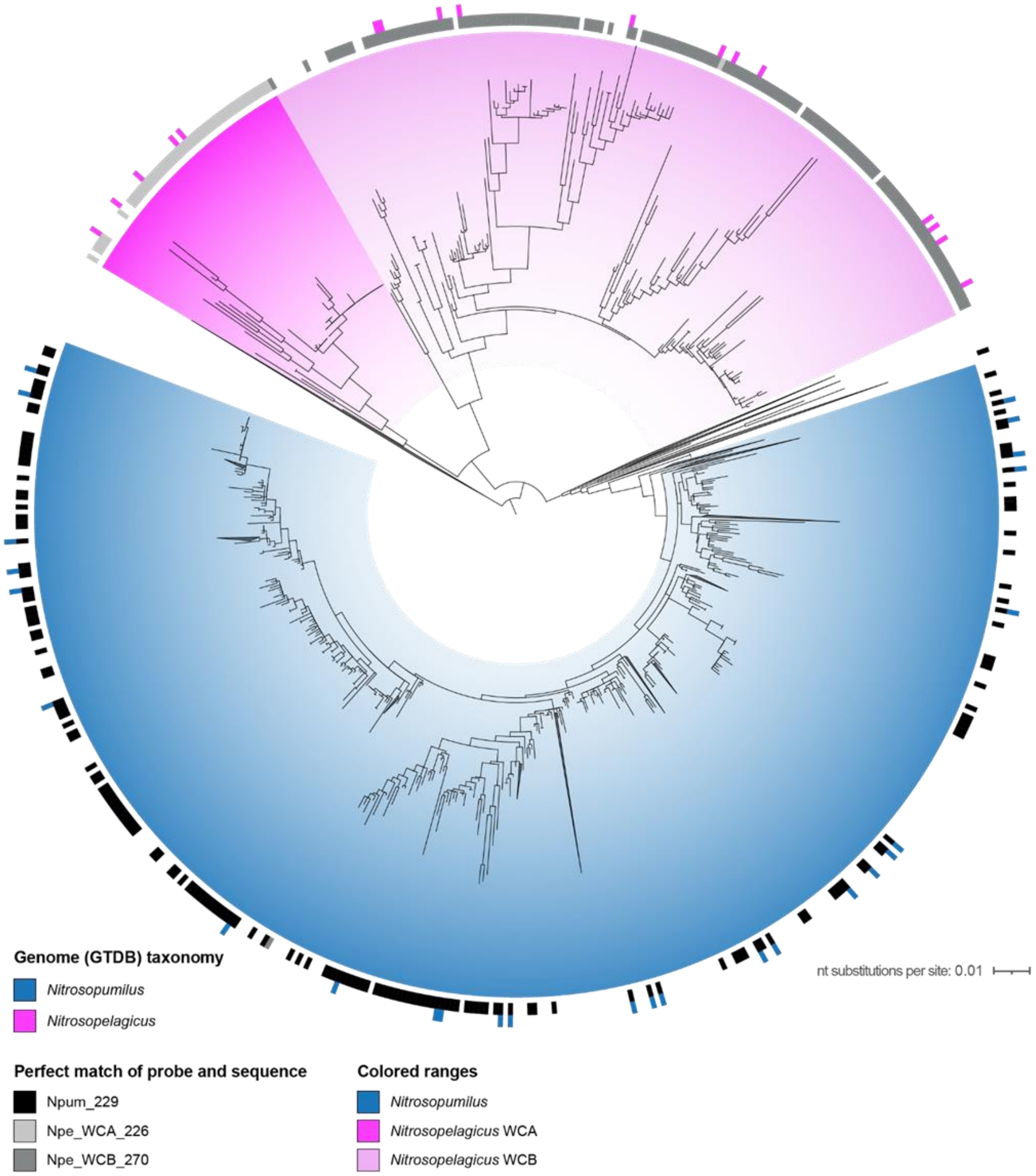

Supplementary Fig. 8: 16S rRNA gene tree of *Nitrosopumilus*, *Nitrosopelagicus* WCA and WCB. Tree includes 16S rRNA gene sequences from SILVA[54] (criteria: *Nitrosopumilales*, sequence length >1399 nucleotides, sequence quality >90, pintail quality >90) and 16S rRNA genes from GTDB/GEM genomes. Tree was constructed using in IQ-TREE2[51], model TVMe+R6, based on a MAFFT (v.7.487)[52] alignment, trimmed using trimal (v1.4.rev15)[53] and re-rooted at *Nitrosotenuis*. Scale bar represents nucleotide substitutions per site. Shading denotes group affiliation, with *Nitrosopelagicus* WCA (magenta) and WCB (rose), and *Nitrosopumilus* (blue). Inner ring shows sequences with perfect match (100% identity across full probe length) to the respective probes (Npum_229 in black, Npe_WCA_226 in light gray,

Npe_WCB_270 in dark gray). Outer ring shows the taxonomic assignment of GTDB genomes that contain 16S rRNA genes (*Nitrosopumilus* in blue and *Nitrosopelagicus* in magenta).

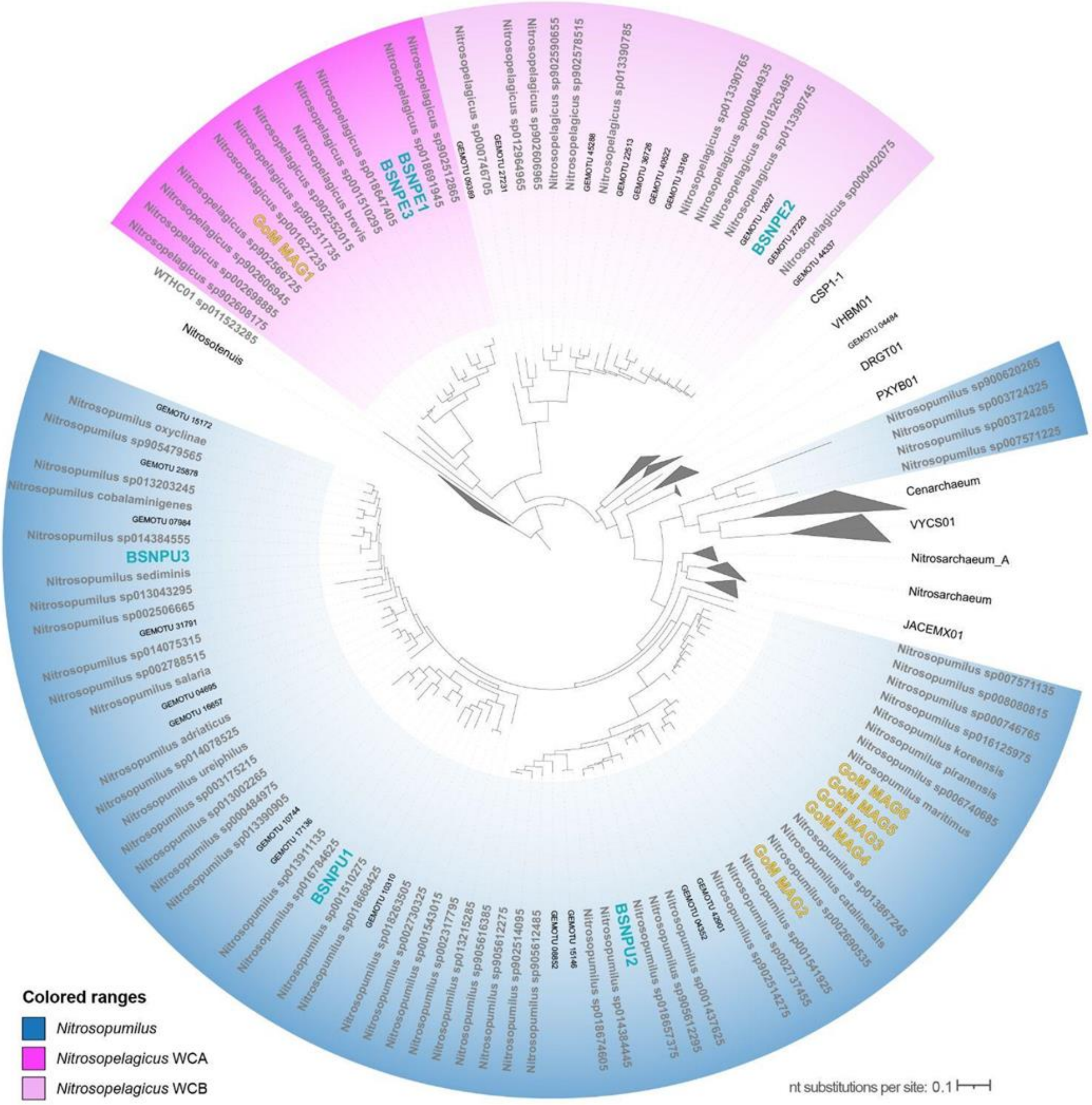

Supplementary Fig. 9: Phylogenetic placement of the AOA MAGs. Sequences from the Gulf of Mexico (yellow) and the Black Sea (turquoise) and reference genomes from GTDB/GEM (with assigned taxonomy, gray) were used for tree calculation. *Nitrosopelagicus* WCA (magenta) and WCB (rose) and *Nitrosopumilus* (blue) clades are indicated. The tree was constructed using anvio-7.1, with sequence alignment using muscle and phylogeny calculation using FastTree 2[34,55,56]. Scale bar represents nucleotide substitutions per site.

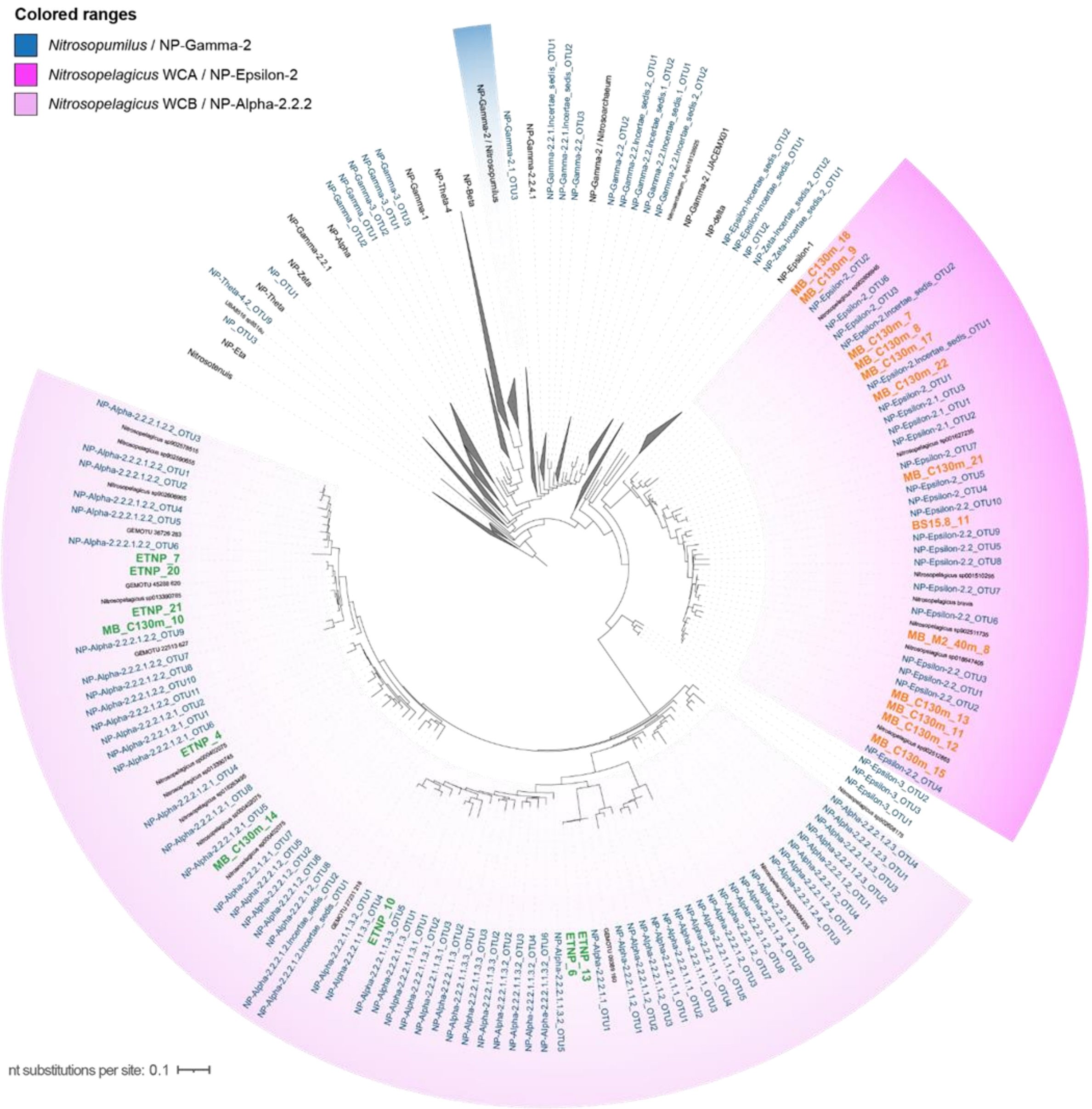

Supplementary Fig. 10: *amoA* phylogenetic tree including GEM/GTDB reference sequences with assigned taxonomy (black), *amoA* references classified by Alves et al.[11] (dark blue), selected WCA (orange) and WCB (green) *amoA* reference sequences from Francis et al.[16]. *Nitrosopelagicus* WCA (magenta), WCB (rose) and *Nitrosopumilus* (blue) clades are indicated. Note that due to the differences in tree calculation and the additional sequences included, the tree topology partly differs from the one reported by Alves et al.[11]. Phylogenetic tree was calculated using IQ-TREE2[51], model GTR+F+R9, based on a MAFFT[52] alignment, trimmed using trimal[53] and re-rooted at *Nitrosotenuis*. Scale bar represents nucleotide substitutions per site.

Supplementary Tables

Supplementary Table 1: $^{15}N^{13}C$-tracers and ^{14}N-pools added for process rate determinations in the Angola Gyre and the Black Sea.

**Environment**	**Investigated process**	**15N-tracer and 14N-pool additions**						**Oxygen**
		**15N-NH4+**	**15N-urea**	**14N-NO2-**	**14N-NH4+**	**14N-N2O**	**13C-DIC**	
Angola Gyre	Ammonia oxidation	200 nM		1 µM				oxic
Angola Gyre	Urea-derived oxidation		100 nM = 200 nM-15N	1 µM				oxic
Angola Gyre	Urea-derived oxidation with ammonium pool		100 nM = 200 nM-15N	1 µM	1 µM			oxic
Black Sea	Ammonia oxidation	5 µM		5 µM		200 nM	240 nM	lower two depths anoxic, upper two depths with 10 µM oxygen
Black Sea	Urea-derived oxidation		1 µM = 2 µM-15N	5 µM		200 nM	240 nM	lower two depths anoxic, upper two depths with 10 µM oxygen
Black Sea	Urea-derived oxidation with ammonium pool		1 µM = 2 µM-15N	5 µM	5 µM	200 nM	240 nM	lower two depths anoxic, upper two depths with 10 µM oxygen
15N-ammonium sulfate (98% 15N), 15N13C-urea (99% 13C, 98% 15N), 13C-DIC (13C-NaHCO3, 99% 13C), all 15N- and 14N- and 13C-compounds were obtained from Sigma								

Supplementary Table 2: Overview CARD-FISH probe specifics

Target Group	Probe Name	Probe Sequence 5' to 3'	Optimal FA concentration [%]	Competitor Name	Competitor Sequence 5' to 3'
Nitrosopumilus	Npum_229	CAACAAACTGATAGGCCGCAGTCCCA	30	Npum_229_c1	CAACAAgCTGATAGGCCGCAGTCCCA
				Npum_229_c2	CtACAAcCTGATAGGCCGCAGcCCCA
				Npum_229_c3	CAACAAcCTGATcGGCCGCAGTCCCA
Nitrosopelagicus WCA	Npe_WCA_226	CTGATAGCTCGCAGTCCCATCC	30	NpeI_226_c1	CTGATGGCTCGCAGCCCCATCC
				NpeI_226_c2	CTGATAGCcCGCAGgCCCATCC
				NpeI_226_c3	CTGATAGgTCGCAGTCCCATCC
Nitrosopelagicus WCB	Npe_WCB_270	CCGTACCTGTAATCGCCTTGGT	20	NpeIII_270_c1	CCGTACCcGTcATCGCCTTGGT
				NpeIII_270_c2	CCGTACCTGTtATCGCCTTGGT

Supplementary Table 3: AOA metagenome-assembled genome (MAG) information from the Black Sea and relative MAG abundances. Completeness, contamination and heterogeneity was calculated using CheckM.

**MAG ID**	**Taxonomy**	**Completeness (%)**	**Contamination (%)**	**Heterogeneity (%)**	**binned *amoA***	**binned *ureC***	**binned *dur3***	**binned 16S**
BSNPU1	*Nitrosopumilus sp.*	94	1.4	0.0	present	present	NA	present
BSNPU2	*Nitrosopumilus sp.*	92.1	5.3	71.43	present	NA	NA	NA
BSNPU3	*Nitrosopumilus sp.*	82.9	1.3	50.0	present	present	present	NA
BSNPE1	*Nitrosopelagicus sp.*	91.26	1.94	0.0	present	present	present	NA
BSNPE2	*Nitrosopelagicus sp.*	88.2	3.9	100.0	present	NA	NA	NA
BSNPE3	*Nitrosopelagicus sp.*	53.9	0.0	0.0	present	NA	NA	NA

Supplementary References

1 Parks, D. H. *et al.* GTDB: an ongoing census of bacterial and archaeal diversity through a phylogenetically consistent, rank normalized and complete genome-based taxonomy. *Nucleic Acids Research* **50**, D785-D794, doi:10.1093/nar/gkab776 (2022).

2 Nayfach, S. *et al.* A genomic catalog of Earth's microbiomes. *Nature Biotechnology* **39**, 499-509, doi:10.1038/s41587-020-0718-6 (2021).

3 Jain, C., Rodriguez-R, L. M., Phillippy, A. M., Konstantinidis, K. T. & Aluru, S. High throughput ANI analysis of 90K prokaryotic genomes reveals clear species boundaries. *Nature Communications* **9**, doi:10.1038/s41467-018-07641-9 (2018).

4 Hyatt, D. *et al.* Prodigal: prokaryotic gene recognition and translation initiation site identification. *BMC Bioinformatics* **11**, 119, doi:10.1186/1471-2105-11-119 (2010).

5 Eren, A. M. *et al.* Community-led, integrated, reproducible multi-omics with anvi'o. *Nature Microbiology* **6**, 3-6, doi:10.1038/s41564-020-00834-3 (2020).

6 Rasko, D. A., Myers, G. S. & Ravel, J. Visualization of comparative genomic analyses by BLAST score ratio. *BMC Bioinformatics* **6**, 2, doi:10.1186/1471-2105-6-2 (2005).

7 Speth, D. R. & Orphan, V. J. Metabolic marker gene mining provides insight in global mcrA diversity and, coupled with targeted genome reconstruction, sheds further light on metabolic potential of the Methanomassiliicoccales. *PeerJ* **6**, e5614, doi:10.7717/peerj.5614 (2018).

8 Geer, L. Y., Domrachev, M., Lipman, D. J. & Bryant, S. H. CDART: Protein Homology by Domain Architecture. *Genome Research* **12**, 1619-1623, doi:10.1101/gr.278202 (2002).

9 Buchfink, B., Reuter, K. & Drost, H.-G. Sensitive protein alignments at tree-of-life scale using DIAMOND. *Nature methods* **18**, 366-368 (2021).

10 Herbold, C. W. *et al.* Ammonia-oxidising archaea living at low pH: Insights from comparative genomics. *Environmental Microbiology* **19**, 4939-4952, doi:10.1111/1462-2920.13971 (2017).

11 Alves, R. J. E., Minh, B. Q., Urich, T., von Haeseler, A. & Schleper, C. Unifying the global phylogeny and environmental distribution of ammonia-oxidising archaea based on amoA genes. *Nat Commun* **9**, 1517, doi:10.1038/s41467-018-03861-1 (2018).

12 Beman, J. M., Popp, B. N. & Francis, C. A. Molecular and biogeochemical evidence for ammonia oxidation by marine Crenarchaeota in the Gulf of California. *The ISME Journal* **2**, 429-441, doi:10.1038/ismej.2007.118 (2008).

13 Santoro, A. E. *et al.* Thaumarchaeal ecotype distributions across the equatorial Pacific Ocean and their potential roles in nitrification and sinking flux attenuation. *Limnology and Oceanography* **62**, 1984-2003, doi:10.1002/lno.10547 (2017).

14 Smith, J. M., Casciotti, K. L., Chavez, F. P. & Francis, C. A. Differential contributions of archaeal ammonia oxidizer ecotypes to nitrification in coastal surface waters. *The ISME Journal* **8**, 1704-1714, doi:10.1038/ismej.2014.11 (2014).

15 Santoro, A. E. & Casciotti, K. L. Enrichment and characterization of ammonia-oxidizing archaea from the open ocean: phylogeny, physiology and stable isotope fractionation. *The ISME Journal* **5**, 1796-1808, doi:10.1038/ismej.2011.58 (2011).

16 Francis, C. A., Roberts, K. J., Beman, J. M., Santoro, A. E. & Oakley, B. B. Ubiquity and diversity of ammonia-oxidizing archaea in water columns and sediments of the ocean. *Proceedings of the National Academy of Sciences* **102**, 14683-14688, doi:10.1073/pnas.0506625102 (2005).

17 Schramm, A., Fuchs, B. M., Nielsen, J. L., Tonolla, M. & Stahl, D. A. Fluorescence in situ hybridization of 16S rRNA gene clones (Clone-FISH) for probe validation and screening of clone libraries. *Environmental Microbiology* **4**, 713-720, doi:10.1046/j.1462-2920.2002.00364.x (2002).

18 Inoue, H., Nojima, H. & Okayama, H. High efficiency transformation of Escherichia coli with plasmids. *Gene* **96**, 23-28, doi:10.1016/0378-1119(90)90336-p (1990).

19 Hanahan, D. Studies on transformation of Escherichia coli with plasmids. *Journal of Molecular Biology* **166**, 557-580, doi:https://doi.org/10.1016/S0022-2836(83)80284-8 (1983).

20 Altschul, S. F., Gish, W., Miller, W., Myers, E. W. & Lipman, D. J. Basic local alignment search tool. *Journal of Molecular Biology* **215**, 403-410, doi:https://doi.org/10.1016/S0022-2836(05)80360-2 (1990).

21 Pernthaler, J., Glöckner, F.-O., Schönhuber, W. & Amann, R. in *Methods in Microbiology* Vol. 30 207-226 (Academic Press, 2001).

22 Manz, W., Amann, R., Ludwig, W., Wagner, M. & Schleifer, K.-H. Phylogenetic Oligodeoxynucleotide Probes for the Major Subclasses of Proteobacteria: Problems and Solutions. *Systematic and Applied Microbiology* **15**, 593-600, doi:10.1016/s0723-2020(11)80121-9 (1992).

23 Daims, H., Stoecker, K. & Wagner, M. in *Molecular Microbial Ecology* (Taylor & Francis, 2005).

24 Bayer, B. *et al.* Nitrosopumilus adriaticus sp. nov. and Nitrosopumilus piranensis sp. nov., two ammonia-oxidizing archaea from the Adriatic Sea and members of the class Nitrososphaeria. *International Journal of Systematic and Evolutionary Microbiology* **69**, 1892-1902, doi:10.1099/ijsem.0.003360 (2019).

25 Daims, H., Lücker, S. & Wagner, M. daime, a novel image analysis program for microbial ecology and biofilm research. *Environmental Microbiology* **8**, 200-213, doi:https://doi.org/10.1111/j.1462-2920.2005.00880.x (2006).

26 Kitzinger, K. *et al.* Cyanate and urea are substrates for nitrification by Thaumarchaeota in the marine environment. *Nat Microbiol* **4**, 234-243, doi:10.1038/s41564-018-0316-2 (2019).

27 Bankevich, A. *et al.* SPAdes: A New Genome Assembly Algorithm and Its Applications to Single-Cell Sequencing. *Journal of Computational Biology* **19**, 455-477, doi:10.1089/cmb.2012.0021 (2012).

28 Peng, Y., Leung, H. C. M., Yiu, S. M. & Chin, F. Y. L. IDBA-UD: a <i>de novo</i> assembler for single-cell and metagenomic sequencing data with highly uneven depth. *Bioinformatics* **28**, 1420-1428, doi:10.1093/bioinformatics/bts174 (2012).

29 Eddy, S. R. Accelerated Profile HMM Searches. *PLoS Computational Biology* **7**, e1002195, doi:10.1371/journal.pcbi.1002195 (2011).

30 Edgar, R. C. Search and clustering orders of magnitude faster than BLAST. *Bioinformatics* **26**, 2460-2461, doi:10.1093/bioinformatics/btq461 (2010).

31 Li, D. *et al.* MEGAHIT v1.0: A fast and scalable metagenome assembler driven by advanced methodologies and community practices. *Methods* **102**, 3-11, doi:https://doi.org/10.1016/j.ymeth.2016.02.020 (2016).

32 CoverM (Centre for Microbiome Research, Queensland University of Technology, 2007).

33 Tamames, J. & Puente-Sanchez, F. SqueezeMeta, A Highly Portable, Fully Automatic Metagenomic Analysis Pipeline. *Front Microbiol* **9**, 3349, doi:10.3389/fmicb.2018.03349 (2018).

34 Eren, A. M. *et al.* Anvi'o: an advanced analysis and visualization platform for 'omics data. *PeerJ* **3**, e1319, doi:10.7717/peerj.1319 (2015).

35 Li, H. Minimap2: pairwise alignment for nucleotide sequences. *Bioinformatics* **34**, 3094-3100, doi:10.1093/bioinformatics/bty191 (2018).

36 Li, H. New strategies to improve minimap2 alignment accuracy. *Bioinformatics* **37**, 4572-4574, doi:10.1093/bioinformatics/btab705 (2021).

37 Danecek, P. *et al.* Twelve years of SAMtools and BCFtools. *GigaScience* **10**, giab008, doi:10.1093/gigascience/giab008 (2021).

38 Shen, W., Le, S., Li, Y. & Hu, F. SeqKit: A Cross-Platform and Ultrafast Toolkit for FASTA/Q File Manipulation. *PLOS ONE* **11**, e0163962, doi:10.1371/journal.pone.0163962 (2016).

39 Prjibelski, A., Antipov, D., Meleshko, D., Lapidus, A. & Korobeynikov, A. Using SPAdes De Novo Assembler. *Current Protocols in Bioinformatics* **70**, doi:10.1002/cpbi.102 (2020).

40 Parks, D. H., Imelfort, M., Skennerton, C. T., Hugenholtz, P. & Tyson, G. W. CheckM: assessing the quality of microbial genomes recovered from isolates, single cells, and metagenomes. *Genome Res* **25**, 1043-1055, doi:10.1101/gr.186072.114 (2015).

41 Zhang, L., Altabet, M. A., Wu, T. & Hadas, O. Sensitive Measurement of NH_4^+ $^{15}N/^{14}N$ ($\delta^{15}NH_4^+$) at Natural Abundance Levels in Fresh and Saltwaters. *Analytical Chemistry* **79**, 5297-5303, doi:10.1021/ac070106d (2007).

42 McIlvin, M. R. & Altabet, M. A. Chemical Conversion of Nitrate and Nitrite to Nitrous Oxide for Nitrogen and Oxygen Isotopic Analysis in Freshwater and Seawater. *Analytical Chemistry* **77**, 5589-5595, doi:10.1021/ac050528s (2005).

43 Frey, C. *et al.* Regulation of nitrous oxide production in low-oxygen waters off the coast of Peru. *Biogeosciences* **17**, 2263-2287, doi:10.5194/bg-17-2263-2020 (2020).

44 Dalsgaard, T., Thamdrup, B., Farías, L. & Revsbech, N. P. Anammox and denitrification in the oxygen minimum zone of the eastern South Pacific. *Limnology and Oceanography* **57**, 1331-1346, doi:10.4319/lo.2012.57.5.1331 (2012).

45 Kitzinger, K. *et al.* Single cell analyses reveal contrasting life strategies of the two main nitrifiers in the ocean. *Nat Commun* **11**, 767, doi:10.1038/s41467-020-14542-3 (2020).

46 Martinez-Perez, C. *et al.* The small unicellular diazotrophic symbiont, UCYN-A, is a key player in the marine nitrogen cycle. *Nat Microbiol* **1**, 16163, doi:10.1038/nmicrobiol.2016.163 (2016).

47 Wan, X. S. *et al.* Significance of Urea in Sustaining Nitrite Production by Ammonia Oxidizers in the Oligotrophic Ocean. *Global Biogeochemical Cycles* **38**, doi:10.1029/2023gb007996 (2024).

48 Bristow, L. A. *et al.* Ammonium and nitrite oxidation at nanomolar oxygen concentrations in oxygen minimum zone waters. *Proc Natl Acad Sci U S A* **113**, 10601-10606, doi:10.1073/pnas.1600359113 (2016).

49 De Brabandere, L., Thamdrup, B., Revsbech, N. P. & Foadi, R. A critical assessment of the occurrence and extend of oxygen contamination during anaerobic incubations utilizing commercially available vials. *Journal of Microbiological Methods* **88**, 147-154, doi:https://doi.org/10.1016/j.mimet.2011.11.001 (2012).

50 Gruber-Vodicka, H. R., Seah, B. K. B. & Pruesse, E. phyloFlash: Rapid Small-Subunit rRNA Profiling and Targeted Assembly from Metagenomes. *mSystems* **5**, doi:10.1128/mSystems.00920-20 (2020).

51 Minh, B. Q. *et al.* IQ-TREE 2: New Models and Efficient Methods for Phylogenetic Inference in the Genomic Era. *Molecular Biology and Evolution* **37**, 1530-1534, doi:10.1093/molbev/msaa015 (2020).

52 Katoh, K. & Standley, D. M. MAFFT Multiple Sequence Alignment Software Version 7: Improvements in Performance and Usability. *Molecular Biology and Evolution* **30**, 772-780, doi:10.1093/molbev/mst010 (2013).

53 Capella-Gutiérrez, S., Silla-Martínez, J. M. & Gabaldón, T. trimAl: a tool for automated alignment trimming in large-scale phylogenetic analyses. *Bioinformatics* **25**, 1972-1973, doi:10.1093/bioinformatics/btp348 (2009).

54 Quast, C. *et al.* The SILVA ribosomal RNA gene database project: improved data processing and web-based tools. *Nucleic Acids Research* **41**, D590-D596, doi:10.1093/nar/gks1219 (2012).

55 Price, M. N., Dehal, P. S. & Arkin, A. P. FastTree 2 – Approximately Maximum-Likelihood Trees for Large Alignments. *PLoS ONE* **5**, e9490, doi:10.1371/journal.pone.0009490 (2010).

56 Edgar, R. C. Muscle5: High-accuracy alignment ensembles enable unbiased assessments of sequence homology and phylogeny. *Nature Communications* **13**, doi:10.1038/s41467-022-34630-w (2022).
