# Supplementary material for: Urea use drives niche separation between dominant marine ammonia oxidizing archaea
Source: Nat Commun. 2025 Dec 6;16:10946. doi: 10.1038/s41467-025-67048-1 (PMC12686405; doi:10.1038/s41467-025-67048-1)
Supplement: Supplementary file 2 — Description of Additional Supplementary Information [file 41467_2025_67048_MOESM2_ESM.pdf]

Description of Additional Supplementary Files

File Name: Supplementary Data 1
Description: CTD data, measured nutrient concentrations, process rates, AOA specific CARDFISH counts and phyloFlash results from the Gulf of Mexico, the Black Sea and the Angola Gyre.

File Name: Supplementary Data 2
Description: Sequencing statistics, custom gene databases (amoA AOA, dur3 and ureC) used for BSR approach and RPKM values of amoA AOA, ureC and dur3 for all samples of the Gulf of Mexico, Black Sea and Angola Gyre.

File Name: Supplementary Data 3
Description: Target and non-target hits of newly designed CARD-FISH probes and competitors and AOA coverage of probes in samples from the Black Sea.

File Name: Supplementary Data 4
Description: Accession numbers of sequences used for 16S rRNA gene, amoA and UreC trees and CARD-FISH probe checks.

File Name: Supplementary Data 5
Description: nanoSIMS measurements from the Gulf of Mexico and the Black Sea.
