# Supplementary material for: Urea use drives niche separation between dominant marine ammonia oxidizing archaea
Source: Nat Commun. 2025 Dec 6;16:10946. doi: 10.1038/s41467-025-67048-1 (PMC12686405; doi:10.1038/s41467-025-67048-1)
Supplement: Supplementary file 8 — Reporting Summary [file 41467_2025_67048_MOESM8_ESM.pdf]

nature portfolio

Corresponding author(s): Hannah K. Marchant

Last updated by author(s): Oct 7, 2025

Reporting Summary

Nature Portfolio wishes to improve the reproducibility of the work that we publish. This form provides structure for consistency and transparency in reporting. For further information on Nature Portfolio policies, see our Editorial Policies and the Editorial Policy Checklist.

Statistics

For all statistical analyses, confirm that the following items are present in the figure legend, table legend, main text, or Methods section.

n/a	Confirmed	
☐	☒	The exact sample size (n) for each experimental group/condition, given as a discrete number and unit of measurement
☐	☒	A statement on whether measurements were taken from distinct samples or whether the same sample was measured repeatedly
☐	☒	The statistical test(s) used AND whether they are one- or two-sided<br>*Only common tests should be described solely by name; describe more complex techniques in the Methods section.*
☒	☐	A description of all covariates tested
☐	☒	A description of any assumptions or corrections, such as tests of normality and adjustment for multiple comparisons
☐	☒	A full description of the statistical parameters including central tendency (e.g. means) or other basic estimates (e.g. regression coefficient) AND variation (e.g. standard deviation) or associated estimates of uncertainty (e.g. confidence intervals)
☐	☒	For null hypothesis testing, the test statistic (e.g. F, t, r) with confidence intervals, effect sizes, degrees of freedom and P value noted<br>*Give P values as exact values whenever suitable.*
☒	☐	For Bayesian analysis, information on the choice of priors and Markov chain Monte Carlo settings
☒	☐	For hierarchical and complex designs, identification of the appropriate level for tests and full reporting of outcomes
☒	☐	Estimates of effect sizes (e.g. Cohen's d, Pearson's r), indicating how they were calculated

Our web collection on statistics for biologists contains articles on many of the points above.

Software and code

Policy information about availability of computer code

Data collection	DIAMOND blastx, blastn, Wincurve (nanoSIMS software), hmmsearch, bbmap, Zeiss Blue, anvi'o v.7.1, NCBI CDART
Data analysis	MS Excel 2016, RStudio v2023.12.1 (with packages dbplyr v2.5.0, tidyr v1.3.1, ggplot2 v3.5.1, patchwork v1.2.0, scales v1.3.0), TrimGalore v0.6.7, Cutadapt v4.0, Python v3.7.12, ARB Silva (SSU database v138.1), GTDB (database v207), GEM (database), fastANI v1.32, Anvi'o v7.1, IQ-TREE2, R, phyloFlash v3.4 and 3.4.1, Look@nanoSIMS v.2023-03-16, MATLAB v.R2021b, daime v. 2.2.3, SPAdes v.3.13.0, IDBA v.1.1.3, IDBA-UD, Prodigal v.2.6.3, UCLUST, MEGAHIT v1.2.9, CoverM v.0.6.1, SqueezeMeta v.1.5.1, minimap2 v.2.24-r1122, samtools v.1.16.1, seqkit v.2.3.0, CheckM v.1.2.2, MAFFT (v.7.487), trimal (v1.4.rev15), QGIS v.3.22.4, Zeiss ZEN blue v.3.2

For manuscripts utilizing custom algorithms or software that are central to the research but not yet described in published literature, software must be made available to editors and reviewers. We strongly encourage code deposition in a community repository (e.g. GitHub). See the Nature Portfolio guidelines for submitting code & software for further information.

Data

Policy information about availability of data

All manuscripts must include a data availability statement. This statement should provide the following information, where applicable:

- Accession codes, unique identifiers, or web links for publicly available datasets
- A description of any restrictions on data availability
- For clinical datasets or third party data, please ensure that the statement adheres to our policy

All sequence data and AOA MAGs generated in this study will be deposited in NCBI under BioProject number: PRJNA1091553. Nutrient Data for the Angola Gyre

cruise has been deposited under https://doi.pangaea.de/10.1594/PANGAEA.931090; nutrient data for the Black Sea is given in Supplementary File 1, for the Gulf of Mexico refer to Kitzinger et al. 2019 (ref. 3). NanoSIMS images and raw data are available upon request.

Research involving human participants, their data, or biological material

Policy information about studies with human participants or human data. See also policy information about sex, gender (identity/presentation), and sexual orientation and race, ethnicity and racism.

Reporting on sex and gender	No human participants were used in this study.
Reporting on race, ethnicity, or other socially relevant groupings	No human participants were used in this study.
Population characteristics	No human participants were used in this study.
Recruitment	No human participants were used in this study.
Ethics oversight	No human participants were used in this study.

Note that full information on the approval of the study protocol must also be provided in the manuscript.

Field-specific reporting

Please select the one below that is the best fit for your research. If you are not sure, read the appropriate sections before making your selection.

☐ Life sciences ☐ Behavioural & social sciences ☒ Ecological, evolutionary & environmental sciences

For a reference copy of the document with all sections, see nature.com/documents/nr-reporting-summary-flat.pdf

Life sciences study design

All studies must disclose on these points even when the disclosure is negative.

Sample size	*Describe how sample size was determined, detailing any statistical methods used to predetermine sample size OR if no sample-size calculation was performed, describe how sample sizes were chosen and provide a rationale for why these sample sizes are sufficient.*
Data exclusions	*Describe any data exclusions. If no data were excluded from the analyses, state so OR if data were excluded, describe the exclusions and the rationale behind them, indicating whether exclusion criteria were pre-established.*
Replication	*Describe the measures taken to verify the reproducibility of the experimental findings. If all attempts at replication were successful, confirm this OR if there are any findings that were not replicated or cannot be reproduced, note this and describe why.*
Randomization	*Describe how samples/organisms/participants were allocated into experimental groups. If allocation was not random, describe how covariates were controlled OR if this is not relevant to your study, explain why.*
Blinding	*Describe whether the investigators were blinded to group allocation during data collection and/or analysis. If blinding was not possible, describe why OR explain why blinding was not relevant to your study.*

Behavioural & social sciences study design

All studies must disclose on these points even when the disclosure is negative.

Study description	*Briefly describe the study type including whether data are quantitative, qualitative, or mixed-methods (e.g. qualitative cross-sectional, quantitative experimental, mixed-methods case study).*
Research sample	*State the research sample (e.g. Harvard university undergraduates, villagers in rural India) and provide relevant demographic information (e.g. age, sex) and indicate whether the sample is representative. Provide a rationale for the study sample chosen. For studies involving existing datasets, please describe the dataset and source.*
Sampling strategy	*Describe the sampling procedure (e.g. random, snowball, stratified, convenience). Describe the statistical methods that were used to predetermine sample size OR if no sample-size calculation was performed, describe how sample sizes were chosen and provide a rationale for why these sample sizes are sufficient. For qualitative data, please indicate whether data saturation was considered, and what criteria were used to decide that no further sampling was needed.*
Data collection	*Provide details about the data collection procedure, including the instruments or devices used to record the data (e.g. pen and paper, computer, eye tracker, video or audio equipment) whether anyone was present besides the participant(s) and the researcher, and whether the researcher was blind to experimental condition and/or the study hypothesis during data collection.*

Timing	*Indicate the start and stop dates of data collection. If there is a gap between collection periods, state the dates for each sample cohort.*
Data exclusions	*If no data were excluded from the analyses, state so OR if data were excluded, provide the exact number of exclusions and the rationale behind them, indicating whether exclusion criteria were pre-established.*
Non-participation	*State how many participants dropped out/declined participation and the reason(s) given OR provide response rate OR state that no participants dropped out/declined participation.*
Randomization	*If participants were not allocated into experimental groups, state so OR describe how participants were allocated to groups, and if allocation was not random, describe how covariates were controlled.*

Ecological, evolutionary & environmental sciences study design

All studies must disclose on these points even when the disclosure is negative.

Study description	We investigated the utilization of urea and ammonium as substrates for marine ammonia oxidizing archaea across three environments of contrasting nutrient availability (Gulf of Mexico, Black Sea, Angola Gyre). Biogeochemical rate measurements on bulk seawater were performed using 15N-labeled ammonium or urea. To assess substrate preferences, we performed 15N urea incubations with and without experimental unlabeled ammonium addition. The AOA diversity and abundance was assessed using metagenomics and metatranscriptomics, and we assessed the prevalence of AOA genes for ammonia and urea utilization. For the Black Sea, we assessed single cell ammonium and urea assimilation for the two most abundant AOA groups, Nitrosopumilus and Nitrosopelagicus WCA, by combining CARD-FISH and nanoSIMS, and compared this data to previous analyses from the Gulf of Mexico.
Research sample	Data from the Gulf of Mexico has been previously published (Kitzinger et al. 2019, https://doi.org/10.1038/s41564-018-0316-2), and was re-analyzed for abundance and diversity of ammonia and urea utilization genes in metagenomes and metatranscriptomes. For the Black Sea and Angola Gyre, bulk ammonia and urea oxidation rates were examined at 5 and 6 stations, respectively, each for 3-4 depths spanning the upper 150m of the water column. For nanoSIMS analyses in the Black Sea, we investigated ammonium and urea assimilation in two co-occurring groups of AOA, Nitrosopumilus and Nitrosopelagicus WCA, identified using group-specific CARD-FISH probes.
Sampling strategy	No statistical methods were used to pre-determine sample-size. For incubation experiments, time course samples were taken and analyzed using either an independently incubated biological replicate per time point (all incubations in the Black Sea and ammonia oxidation incubations in the Angola Gyre), subsampling from biological triplicates (Gulf of Mexico), or biological duplicates (Urea oxidation rates in the Angola Gyre). Metagenomes and metatranscriptomes were sequenced for multiple stations and depths in single replicates from all environments. nanoSIMS analyses were performed on pooled triplicate samples (Gulf of Mexico) or separately incubated biological replicates (Black Sea), for each group of target cells, statistical analyses were performed to ensure sufficient cells had been analyzed per group for robust conclusions according to Svéden et al 2015 (https://doi.org/10.1093/femsec/fiv131).
Data collection	Bulk seawater samples for nucleic acid extraction, incubations and single cell analyses were collected using Niskin rosettes (Gulf of Mexico, Angola Gyre) or pump-cast CTDs (Black Sea) by H.K.Marchant, J.S.Graf, G.Lavik, J.Milucka, S.Schorn (Angola Gyre) and K.Kitzinger, H.K.Marchant, J. Graf, J.N.v.Arx, J.Milucka (Black Sea). Gas chromatography isotope-ratio mass spectrometry (GC-IRMS) samples were prepared and analyzed by J.Stuehrenberg, K.Kitzinger, G.Lavik. GC-IRMS samples were measured by G.Klockgether and N.Rujanski without prior knowledge on incubation setup and corresponding sample code. NanoSIMS analyses were performed by J. Stuehrenberg and S.Littmann. Bioinformatic analyses were done by J.Stuehrenberg, D.R.Speth and K.Kitzinger. Metadata for sample collection and incubation in the field was recorded in lab books, CTD data on recorded with attached computers and saved on several hard drives/servers. All nanoSIMS, bioinformatics and GC-IRMS data was saved and archived on backed-up servers.
Timing and spatial scale	We investigated three regions of differing nutrient availability, ranging from eutrophic (Gulf of Mexico, Kitzinger et al 2019 https://doi.org/10.1038/s41564-018-0316-2) to mesotrophic (Black Sea) and oligotrophic (Angola Gyre).<br>Six stations in the Western Basin of the Black Sea were sampled between 42° 27.05’N 29°15’W and 44° 12.8’N 30°59.6’W during cruise POS539 with the R/V Poseidon, from November 6th to November 21st 2019. Water was sampled down to 150 m. Depth profile measurements for nutrients were taken at six stations (W1, W2, W3, S2, S3, P1). Stable isotope incubations were carried out at all six stations, and molecular and FISH analyses were done at two stations (S3, W3).<br>Six stations in the Angola Gyre were sampled during cruise M148-2 with the R/V Meteor between 13° 22.799' S, 003° 51.407' E and 00° 39.888' N, 008° 44.839' W from July 2nd to 20th 2018. Samples for nutrient concentration measurements were taken at all stations (doi: 10.1594/PANGAEA.931090). Samples for rate measurements and molecular analyses were collected from three to four depths down to 150 m at stations 210, 211, 213, 214, 215.<br>We sampled down to 150m depths for both the Angola Gyre and the Black Sea cruises to target waters with expected high nitrification rates. In the Angola Gyre, this corresponded to sampling down to below the primary nitrite maximum, in the Black Sea, this corresponded to the region of non-sulfidic waters.
Data exclusions	No data was excluded from analysis. For some experiments, a duplicate incubation set was lost (Angola Gyre urea + ammonium incubations at Station 210, 211, 213 and 214) because the GC-IRMS run failed, and not sufficient sample for remeasurement was available.
Reproducibility	All performed experiments are reported.
Randomization	Mass spectrometric samples were measured by technical staff unaware of incubation type and sample code.
Blinding	Mass spectrometric samples were measured by technical staff unaware of incubation type and sample code.

Did the study involve field work? [x] Yes [ ] No

Field work, collection and transport

Field conditions	Weather conditions on all three cruises were mild. Water parameters are described in detail in the supplementary material of the manuscript.
Location	Field samples were obtained from:<br>- Western Basin of the Black Sea (between 42° 27.05'N 29°15'W and 44° 12.8'N 30°59.6'W) during cruise POS539 with the R/V Poseidon, from November 6th to November 21st 2019. Seawater above the sulfidic layer was collected with a pump cast conductivity temperature depth system (pCTD) equipped with sensors for temperature and salinity (Seabird SBE49 FastCAT), fluorescence (Turner Designs Cyclops-7F), high- (Pyroscience FSO2-Subport) and low-sensitivity oxygen concentrations (Aanderaa optode 4831). Water was sampled down to 150 m. Depth profile measurements for nutrients were taken at six stations (W1, W2, W3, S2, S3, P1). Stable isotope incubations were carried out at all six stations, and molecular and FISH analyses were done at two stations (S3, W3).<br>- Angola Gyre during cruise M148-2 with the R/V Meteor from July 2nd to 20th 2018. Seawater was collected using a CTD rosette equipped with 21x 10L Niskin bottles at all stations (station 210 – 215). Samples for nutrient concentration measurements were taken at all stations (doi: 10.1594/PANGAEA.931090) from profiling CTD casts. Samples for rate measurements and molecular analyses were collected from a separate CTD cast from three to four depths from the ocean surface down to 150 m at stations in the open ocean (Angola Gyre) transect (stations 210, 211, 213, 214, 215).<br>- Northern Gulf of Mexico during cruise PE17-02 with the R/V Pelican, from July 23rd to August 1st, 2016, and is described in Kitzinger et al. 2019, doi:10.1038/s41564-018-0316-2
Access & import/export	All sampling and import permits have been acquired prior to the cruises. No permits under the Nagoya Protocol were required; Gulf of Mexico samples were obtained from waters within the US economic zone, which is not a member to the Nagoya Protocol.<br>Angola Gyre samples were obtained from sites outside national jurisdiction (high sea). The conducted analyses of the microbial community in the Black Sea are excempt from Nagoya Protocol regulations of Romania.
Disturbance	We obtained water samples with clean sampling bottles / tubes at all locations and do not expect any disturbance on the habitat.

Reporting for specific materials, systems and methods

We require information from authors about some types of materials, experimental systems and methods used in many studies. Here, indicate whether each material, system or method listed is relevant to your study. If you are not sure if a list item applies to your research, read the appropriate section before selecting a response.

Materials & experimental systems

n/a	Involved in the study
[x]	[ ] Antibodies
[x]	[ ] Eukaryotic cell lines
[x]	[ ] Palaeontology and archaeology
[x]	[ ] Animals and other organisms
[x]	[ ] Clinical data
[x]	[ ] Dual use research of concern
[x]	[ ] Plants

Methods

n/a	Involved in the study
[x]	[ ] ChIP-seq
[x]	[ ] Flow cytometry
[x]	[ ] MRI-based neuroimaging

Antibodies

Antibodies used	*Describe all antibodies used in the study; as applicable, provide supplier name, catalog number, clone name, and lot number.*
Validation	*Describe the validation of each primary antibody for the species and application, noting any validation statements on the manufacturer's website, relevant citations, antibody profiles in online databases, or data provided in the manuscript.*

Eukaryotic cell lines

Policy information about cell lines and Sex and Gender in Research

Cell line source(s)	*State the source of each cell line used and the sex of all primary cell lines and cells derived from human participants or vertebrate models.*
Authentication	*Describe the authentication procedures for each cell line used OR declare that none of the cell lines used were authenticated.*
Mycoplasma contamination	*Confirm that all cell lines tested negative for mycoplasma contamination OR describe the results of the testing for mycoplasma contamination OR declare that the cell lines were not tested for mycoplasma contamination.*

Commonly misidentified lines (See ICLAC register)

Name any commonly misidentified cell lines used in the study and provide a rationale for their use.

Palaeontology and Archaeology

Specimen provenance

Provide provenance information for specimens and describe permits that were obtained for the work (including the name of the issuing authority, the date of issue, and any identifying information). Permits should encompass collection and, where applicable, export.

Specimen deposition

Indicate where the specimens have been deposited to permit free access by other researchers.

Dating methods

If new dates are provided, describe how they were obtained (e.g. collection, storage, sample pretreatment and measurement), where they were obtained (i.e. lab name), the calibration program and the protocol for quality assurance OR state that no new dates are provided.

☐ Tick this box to confirm that the raw and calibrated dates are available in the paper or in Supplementary Information.

Ethics oversight

Identify the organization(s) that approved or provided guidance on the study protocol, OR state that no ethical approval or guidance was required and explain why not.

Note that full information on the approval of the study protocol must also be provided in the manuscript.

Animals and other research organisms

Policy information about studies involving animals; ARRIVE guidelines recommended for reporting animal research, and Sex and Gender in Research

Laboratory animals

For laboratory animals, report species, strain and age OR state that the study did not involve laboratory animals.

Wild animals

Provide details on animals observed in or captured in the field; report species and age where possible. Describe how animals were caught and transported and what happened to captive animals after the study (if killed, explain why and describe method; if released, say where and when) OR state that the study did not involve wild animals.

Reporting on sex

Indicate if findings apply to only one sex; describe whether sex was considered in study design, methods used for assigning sex. Provide data disaggregated for sex where this information has been collected in the source data as appropriate; provide overall numbers in this Reporting Summary. Please state if this information has not been collected. Report sex-based analyses where performed, justify reasons for lack of sex-based analysis.

Field-collected samples

For laboratory work with field-collected samples, describe all relevant parameters such as housing, maintenance, temperature, photoperiod and end-of-experiment protocol OR state that the study did not involve samples collected from the field.

Ethics oversight

Identify the organization(s) that approved or provided guidance on the study protocol, OR state that no ethical approval or guidance was required and explain why not.

Note that full information on the approval of the study protocol must also be provided in the manuscript.

Clinical data

Policy information about clinical studies

All manuscripts should comply with the ICMJE guidelines for publication of clinical research and a completed CONSORT checklist must be included with all submissions.

Clinical trial registration

Provide the trial registration number from ClinicalTrials.gov or an equivalent agency.

Study protocol

Note where the full trial protocol can be accessed OR if not available, explain why.

Data collection

Describe the settings and locales of data collection, noting the time periods of recruitment and data collection.

Outcomes

Describe how you pre-defined primary and secondary outcome measures and how you assessed these measures.

Dual use research of concern

Policy information about dual use research of concern

Hazards

Could the accidental, deliberate or reckless misuse of agents or technologies generated in the work, or the application of information presented in the manuscript, pose a threat to:

No	Yes	
☐	☐	Public health
☐	☐	National security
☐	☐	Crops and/or livestock
☐	☐	Ecosystems
☐	☐	Any other significant area

Experiments of concern

Does the work involve any of these experiments of concern:

No	Yes	
☐	☐	Demonstrate how to render a vaccine ineffective
☐	☐	Confer resistance to therapeutically useful antibiotics or antiviral agents
☐	☐	Enhance the virulence of a pathogen or render a nonpathogen virulent
☐	☐	Increase transmissibility of a pathogen
☐	☐	Alter the host range of a pathogen
☐	☐	Enable evasion of diagnostic/detection modalities
☐	☐	Enable the weaponization of a biological agent or toxin
☐	☐	Any other potentially harmful combination of experiments and agents

Plants

Seed stocks: not applicable.

Novel plant genotypes: not applicable.

Authentication: not applicable.

ChIP-seq

Data deposition

☐ Confirm that both raw and final processed data have been deposited in a public database such as GEO.

☐ Confirm that you have deposited or provided access to graph files (e.g. BED files) for the called peaks.

Data access links
May remain private before publication.: *For "Initial submission" or "Revised version" documents, provide reviewer access links. For your "Final submission" document, provide a link to the deposited data.*

Files in database submission: *Provide a list of all files available in the database submission.*

Genome browser session (e.g. UCSC): *Provide a link to an anonymized genome browser session for "Initial submission" and "Revised version" documents only, to enable peer review. Write "no longer applicable" for "Final submission" documents.*

Methodology

Replicates: *Describe the experimental replicates, specifying number, type and replicate agreement.*

Sequencing depth: *Describe the sequencing depth for each experiment, providing the total number of reads, uniquely mapped reads, length of reads and whether they were paired- or single-end.*

Antibodies: *Describe the antibodies used for the ChIP-seq experiments; as applicable, provide supplier name, catalog number, clone name, and lot number.*

Peak calling parameters: *Specify the command line program and parameters used for read mapping and peak calling, including the ChIP, control and index files used.*

Data quality: *Describe the methods used to ensure data quality in full detail, including how many peaks are at FDR 5% and above 5-fold enrichment.*

Software	*Describe the software used to collect and analyze the ChIP-seq data. For custom code that has been deposited into a community repository, provide accession details.*

Flow Cytometry

Plots

Confirm that:

- [ ] The axis labels state the marker and fluorochrome used (e.g. CD4-FITC).
- [ ] The axis scales are clearly visible. Include numbers along axes only for bottom left plot of group (a 'group' is an analysis of identical markers).
- [ ] All plots are contour plots with outliers or pseudocolor plots.
- [ ] A numerical value for number of cells or percentage (with statistics) is provided.

Methodology

Sample preparation	*Describe the sample preparation, detailing the biological source of the cells and any tissue processing steps used.*
Instrument	*Identify the instrument used for data collection, specifying make and model number.*
Software	*Describe the software used to collect and analyze the flow cytometry data. For custom code that has been deposited into a community repository, provide accession details.*
Cell population abundance	*Describe the abundance of the relevant cell populations within post-sort fractions, providing details on the purity of the samples and how it was determined.*
Gating strategy	*Describe the gating strategy used for all relevant experiments, specifying the preliminary FSC/SSC gates of the starting cell population, indicating where boundaries between "positive" and "negative" staining cell populations are defined.*

- [ ] Tick this box to confirm that a figure exemplifying the gating strategy is provided in the Supplementary Information.

Magnetic resonance imaging

Experimental design

Design type	*Indicate task or resting state; event-related or block design.*
Design specifications	*Specify the number of blocks, trials or experimental units per session and/or subject, and specify the length of each trial or block (if trials are blocked) and interval between trials.*
Behavioral performance measures	*State number and/or type of variables recorded (e.g. correct button press, response time) and what statistics were used to establish that the subjects were performing the task as expected (e.g. mean, range, and/or standard deviation across subjects).*

Acquisition

Imaging type(s)	*Specify: functional, structural, diffusion, perfusion.*
Field strength	*Specify in Tesla*
Sequence & imaging parameters	*Specify the pulse sequence type (gradient echo, spin echo, etc.), imaging type (EPI, spiral, etc.), field of view, matrix size, slice thickness, orientation and TE/TR/flip angle.*
Area of acquisition	*State whether a whole brain scan was used OR define the area of acquisition, describing how the region was determined.*

Diffusion MRI ☐ Used ☐ Not used

Preprocessing

Preprocessing software	*Provide detail on software version and revision number and on specific parameters (model/functions, brain extraction, segmentation, smoothing kernel size, etc.).*
Normalization	*If data were normalized/standardized, describe the approach(es): specify linear or non-linear and define image types used for transformation OR indicate that data were not normalized and explain rationale for lack of normalization.*
Normalization template	*Describe the template used for normalization/transformation, specifying subject space or group standardized space (e.g. original Talairach, MNI305, ICBM152) OR indicate that the data were not normalized.*
Noise and artifact removal	*Describe your procedure(s) for artifact and structured noise removal, specifying motion parameters, tissue signals and physiological signals (heart rate, respiration).*

Volume censoring | *Define your software and/or method and criteria for volume censoring, and state the extent of such censoring.*

Statistical modeling & inference

Model type and settings | *Specify type (mass univariate, multivariate, RSA, predictive, etc.) and describe essential details of the model at the first and second levels (e.g. fixed, random or mixed effects; drift or auto-correlation).*

Effect(s) tested | *Define precise effect in terms of the task or stimulus conditions instead of psychological concepts and indicate whether ANOVA or factorial designs were used.*

Specify type of analysis: ☐ Whole brain ☐ ROI-based ☐ Both

Statistic type for inference | *Specify voxel-wise or cluster-wise and report all relevant parameters for cluster-wise methods.*

(See Eklund et al. 2016)

Correction | *Describe the type of correction and how it is obtained for multiple comparisons (e.g. FWE, FDR, permutation or Monte Carlo).*

Models & analysis

n/a	Involved in the study
☐	☐ Functional and/or effective connectivity
☐	☐ Graph analysis
☐	☐ Multivariate modeling or predictive analysis

Functional and/or effective connectivity | *Report the measures of dependence used and the model details (e.g. Pearson correlation, partial correlation, mutual information).*

Graph analysis | *Report the dependent variable and connectivity measure, specifying weighted graph or binarized graph, subject- or group-level, and the global and/or node summaries used (e.g. clustering coefficient, efficiency, etc.).*

Multivariate modeling and predictive analysis | *Specify independent variables, features extraction and dimension reduction, model, training and evaluation metrics.*
